# General Distributed Neural Control and Sensory Adaptation for Self-Organized Locomotion and Fast Adaptation to Damage of Walking Robots

**DOI:** 10.3389/fncir.2020.00046

**Published:** 2020-08-17

**Authors:** Aitor Miguel-Blanco, Poramate Manoonpong

**Affiliations:** ^1^Embodied Artificial Intelligence and Neurorobotics Lab, SDU Biorobotics, The Maersk Mc-Kinney Møller Institute, University of Southern Denmark, Odense, Denmark; ^2^Bio-Inspired Robotics and Neural Engineering Lab, School of Information Science and Technology, Vidyasirimedhi Institute of Science and Technology, Rayong, Thailand

**Keywords:** synaptic plasticity, legged robot control, neural circuits, walking machines, lesion-induced plasticity, forward model, central pattern generator, serotonin

## Abstract

Walking animals such as invertebrates can effectively perform self-organized and robust locomotion. They can also quickly adapt their gait to deal with injury or damage. Such a complex achievement is mainly performed via coordination between the legs, commonly known as interlimb coordination. Several components underlying the interlimb coordination process (like distributed neural control circuits, local sensory feedback, and body-environment interactions during movement) have been recently identified and applied to the control systems of walking robots. However, while the sensory pathways of biological systems are plastic and can be continuously readjusted (referred to as sensory adaptation), those implemented on robots are typically static. They first need to be manually adjusted or optimized offline to obtain stable locomotion. In this study, we introduce a fast learning mechanism for online sensory adaptation. It can continuously adjust the strength of sensory pathways, thereby introducing flexible plasticity into the connections between sensory feedback and neural control circuits. We combine the sensory adaptation mechanism with distributed neural control circuits to acquire the adaptive and robust interlimb coordination of walking robots. This novel approach is also general and flexible. It can automatically adapt to different walking robots and allow them to perform stable self-organized locomotion as well as quickly deal with damage within a few walking steps. The adaptation of plasticity after damage or injury is considered here as lesion-induced plasticity. We validated our adaptive interlimb coordination approach with continuous online sensory adaptation on simulated 4-, 6-, 8-, and 20-legged robots. This study not only proposes an adaptive neural control system for artificial walking systems but also offers a possibility of invertebrate nervous systems with flexible plasticity for locomotion and adaptation to injury.

## 1. Introduction

Walking animals show robust and adaptive locomotion. They can form their gaits in a self-organized manner as well as quickly adapt to environmental and body changes, including damage (Wolf and Büschges, 1997; Büschges and Manira, 1998; Grabowska et al., 2012). This complex ability is achieved through adaptive interlimb coordination mechanisms. Biological investigation reveals that the adaptive coordination in walking animals is largely attained by distributed neural control mechanisms with central pattern generators (CPGs), local or proprioceptive feedback, and body dynamics (Pearson and Iles, 1970, 1973; Bässler and Wegner, 1983; Dean, 1989; Berkowitz and Laurent, 1996).

While these components have been identified, their details have not been fully applied to artificial walking systems. For example, while animal experiments show that synaptic connections in sensory motor pathways are plastic (i.e., sensory adaptation) (Whelan and Pearson, 1997; Wolf and Büschges, 1997; Wark et al., 2007) to allow for stable locomotion and adaptation, this plasticity with continuous synaptic changes has been largely ignored in robotic implementation. Typically, the connections between sensory feedback and neural circuits for locomotion control of walking robots are static. To obtain stable locomotion, these connections are usually adjusted manually, or empirically chosen, for specific walking robots (Owaki et al., 2012; Barikhan et al., 2014). In some cases, machine learning techniques are employed first to optimize the connections through simulation before implementing them on real robots (Hwangbo et al., 2019). Accordingly, unexpected situations such as leg damage might lead to unstable locomotion if the sensory connection strength cannot be automatically or continuously adjusted to deliver proper information for adaptation. Furthermore, transferring the control system with the tuned or optimized connections from one walking robot to another might not work effectively.

**Figure 1:**
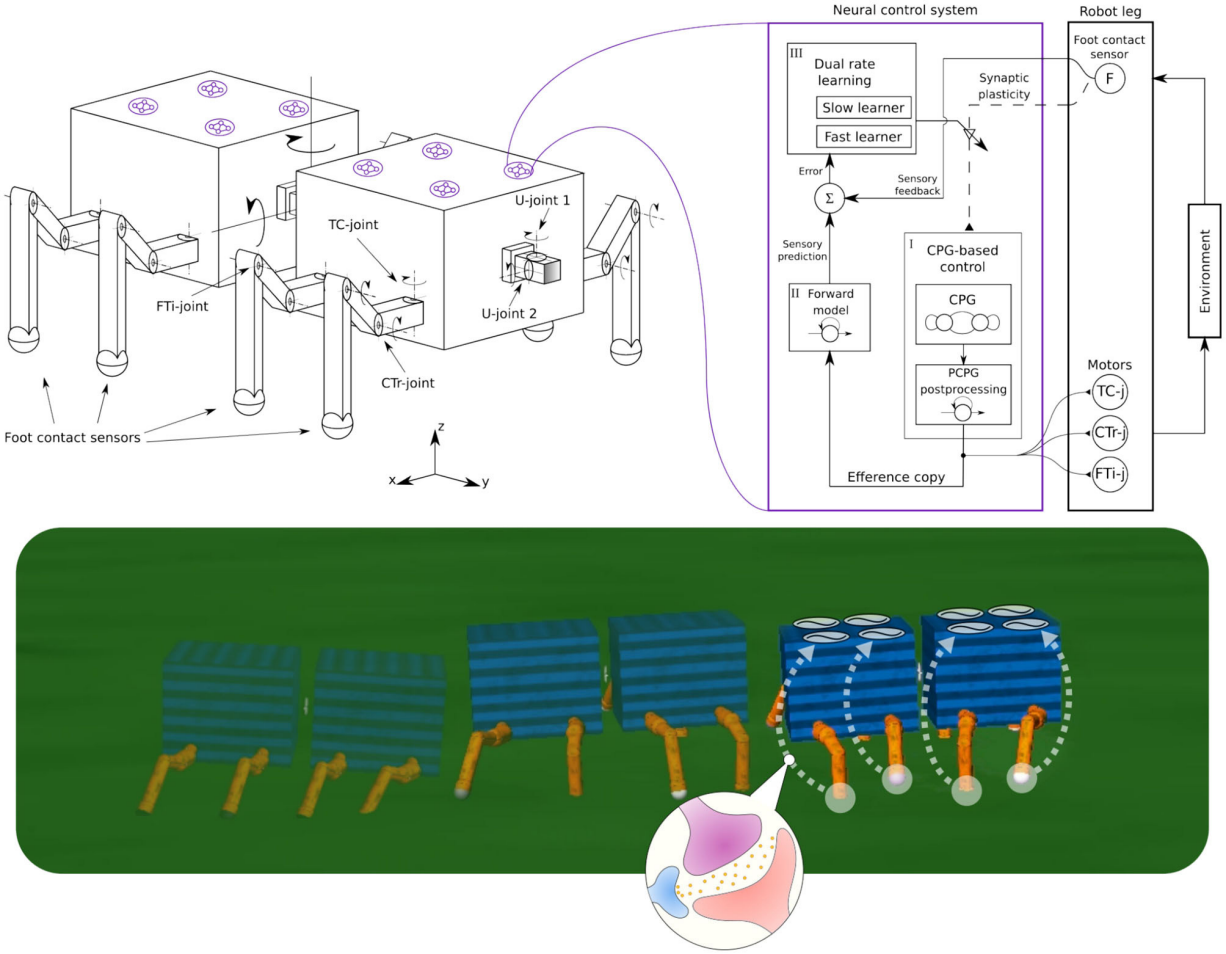
**(Left)** The robot structure for our experiments, consisting of multiple invertebrate-inspired body segments where each has either one or two pairs of identical legs. Legs are inspired by insect limbs and have three joints: Thoraco-coxal (TC-), coxa-trochanteral (CTr-), and femur-tibia (FTi-) joints. The TC-joint enables forward (+) and backward (−) movement. The CTr- joint enables elevation (+) and depression (−) of the leg. The FTi-joint enables extension (+) and flexion (−) of one leg. The connection between body segments is performed by a universal passive connector with two joints(U-joint1 and U-joint2) and limited rotation. **(Right)** A complete adaptive neural control system for each leg, consisting of (i) CPG-based control, (ii) forward model, and (iii) dual rate learning. This is a distributed and decoupled control structure where each leg is driven by one control system and there is no coupling between the control systems. This system uses foot contact or load sensing feedback to alter the CPG phase of each leg; thereby forming interlimb coordination. The CPG post-processing unit (PCPG) converts the CPG outputs into proper motor commands, which are at the same time copied (efference copy) to the forward model for foot contact sensory prediction. The dual rate learning mechanism affects the CPG inputs (i.e., sensory feedback) by adjusting the synaptic plasticity (i.e., sensory feedback connection, see dashed line) based on an error signal between the prediction and actual foot contact feedback (see also Figure 5). **(Bottom)** Self-organized locomotion and adaptation of a walking robot under the adaptive neural control system with sensory synaptic plasticity.

From this perspective, in this study, we introduce a fast learning mechanism for continuous online adaptation or flexible plasticity in sensory pathways (i.e., synaptic connection strength plasticity of sensory feedback) in order to (i) generate stable self-organized locomotion, (ii) deal with damage (known as lesion-induced plasticity), and (iii) be able to automatically adapt to different walking robots. Specifically, the learning mechanism will continuously adjust the connection strength between proprioceptive feedback (i.e., foot contact feedback) and distributed neural CPG-based control circuits (Figure 1). This approach combines bio-inspired key ingredients including: (1) distributed neural CPG-based control circuits without inter-circuit connections for flexible and independent individual leg control, (2) a learning mechanism for proprioceptive sensory adaptation, and (3) body-environment interaction, to acquire adaptive and flexible interlimb coordination for walking robots. This novel approach has more advantages compared to others (Ijspeert et al., 2007; Manoonpong et al., 2008, 2013; Inagaki et al., 2010; Asif, 2012; Ambe et al., 2013) in the following aspects:

- It does not require predefined interlimb coordination (i.e., hardwired neural connections between the CPG circuits), predefined or preoptimized connection strength in sensory pathways, or even the robot's kinematic model.
- It can be directly applied to different walking robots (i.e., generalization and transferability) allowing them to quickly perform stable, self-organized locomotion.
- It can quickly deal with damage within a few walking steps.

We validated our proposed adaptive interlimb coordination approach on simulated 4-, 6-, 8-, and 20-legged robots. We believe that the study pursued here will also sharpen our understanding of how continuous online sensory adaptation with flexible plasticity can be realized and combined with control mechanisms for self-organized locomotion and fast adaptation to damage in walking systems which could not be realized solely by conventional bio-inspired control methods (Espenschied et al., 1996; Ijspeert et al., 2007; Manoonpong et al., 2008, 2013; Inagaki et al., 2010; Asif, 2012; Ambe et al., 2013; Bjelonic et al., 2016) or machine learning techniques (Bongard et al., 2006; Cully et al., 2015; Hwangbo et al., 2019), or their combination (like CPG-based control with reinforcement learning, Ishige et al., 2019) (see section 5 for more details).

## 2. Materials and Methods

In this section, we describe the development of our neural control system for self-organized locomotion and fast adaptation to damage of walking robots. The system exploits neural dynamics and plasticity, proprioceptive feedback (i.e., load sensing feedback), and robot body dynamics to adaptively coordinate robot limbs, called adaptive interlimb coordination. The proposed control system involves three main components: (1) neural CPG-based control with load sensing feedback for rhythmic movement generation, (2) a forward model for sensory prediction, and (3) a dual rate learning mechanism for continuous online sensory adaptation or synaptic connection strength plasticity of sensory feedback (Figure 1). Each of which is described in detail below.

In this setup, each leg is driven by one control system (i.e., one neural CPG-based control circuit, one forward model, and one dual rate learning mechanism). As a consequence, controlling 4-, 6-, 8-, and 20-legged robots will require 4, 6, 8, and 20 neural control systems. For flexibility, we do not define any connection or coupling between the neural control systems. Instead, the coordination among them is mainly achieved by the interaction between the robot and environment, resulting in self-organized locomotion.

Similar configurations have been used before, showing that it is possible to use sensory feedback to adjust the phase between the legs of a walking robot (Owaki et al., 2012; Barikhan et al., 2014). The feedback-based phase adjustment eventually forms a walking pattern, allowing the robot to walk, while the neural CPGs continue to produce the oscillatory signals driving the leg movements during each step. However, this does not guarantee that the robot will always keep a stable gait during walking, since a change in the sensory feedback can appear as a consequence of structural changes in the robot (e.g., leg damage and different configurations). Thus, sufficient sensory feedback information should be maintained to adapt to a new stable pattern. The mechanism for automatic adjustment of sensory feedback strength (or synaptic connection strength plasticity) on the control loop adds a degree of flexibility to the feedback-based phase adjustment method, allowing for adaptation to the changes. It is also important to note that this control does not cover the trajectory of a step. In this case, the individual leg trajectories (i.e., intralimb coordination) have been predefined to fit the normal movement of these limbs based on that of insect legs. However, to adapt the proposed mechanism to other configurations, it is necessary to create an appropriate translation from CPG outputs to motor commands. For example, the robot legs here include three joints, allowing the movement of their tips with respect to the defined trajectory for self-organized locomotion (see section 3).

### 2.1. Neural CPG-Based Control With Load Sensing Feedback

**Figure 2:**
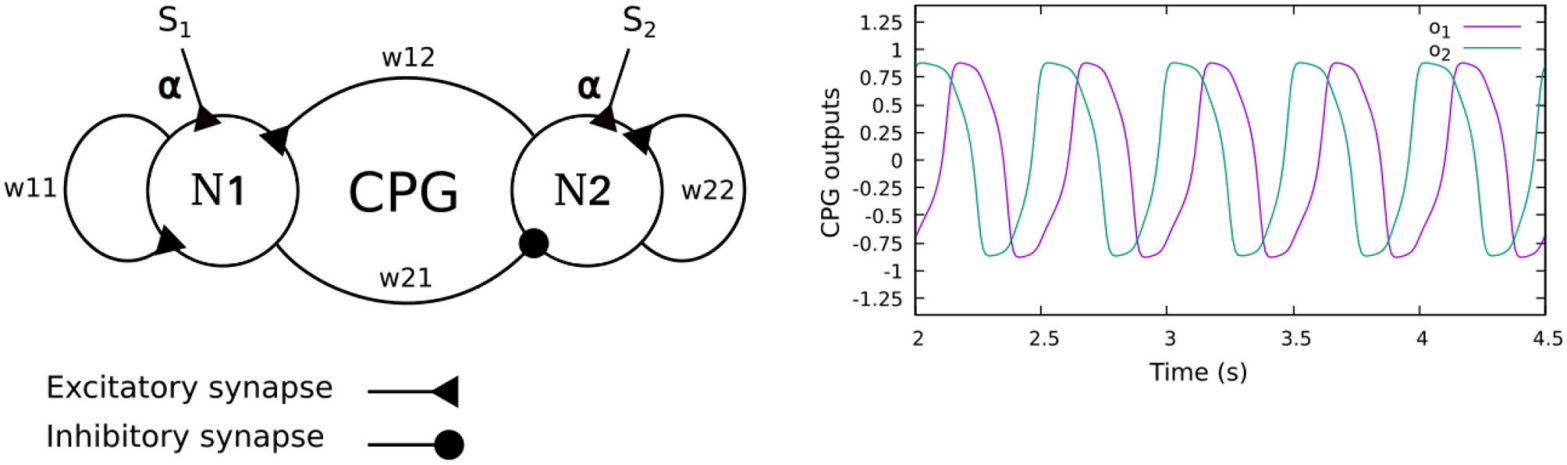
**(Left)** The CPG model of the neural CPG-based control, consisting of two recurrent neurons (N1, N2) with mutual connections. The sensory feedback (*S*_1,2_) is projected to the CPG as its neural inputs. **(Right)** The CPG output signals. The signals differ in phase by π/2 and are further shaped by a CPG postprocessing unit to obtain smooth signals for motor control. In this study, the weights of all CPGs are set to *w*_11_ = 1.4, *w*_22_ = 1.4, *w*_21_ = −0.78, and *w*_12_ = 0.78. With these parameters, the CPG generates periodic signals with a frequency of 1.4 Hz.

The neural CPG-based control is based on two coupled recurrent neurons which generate two periodic signals (Figure 2). The signals are converted into motor commands for driving the leg joints (TC-, CTr-, and FTi-joints) through the CPG postprocessing unit. To automatically adjust the phase of the CPG for interlimb coordination, we use load sensing feedback, projecting to the CPG as its inputs. This technique was proposed by Owaki et al. (2012) and Barikhan et al. (2014). This feedback produces the appropriate phase shifts between the legs to generate a robot walking gait while keeping an understandable and simple control mechanism.

The neurons of the CPG control network are modeled as discrete-time non-spiking neurons. They are updated using a frequency of approximately 27 Hz. The activity of each neuron develops according to:

(1) $$\begin{array}{l} a_{1}\left(t+1\right)=\overset{2}{\underset{j=1}{\sum }}w_{1j}o_{j}\left(t\right)+\alpha S_{1}\left(t\right) \end{array}$$

(2) $$\begin{array}{l} a_{2}\left(t+1\right)=\overset{2}{\underset{j=1}{\sum }}w_{2j}o_{j}\left(t\right)+\alpha S_{2}\left(t\right) \end{array}$$

(3) $$\begin{array}{l} o_{i}\left(t+1\right)=tanh\left(a_{i}\left(t+1\right)\right)\;i=1,2 \end{array}$$

where *w*_1*j*,2*j*_ are the synaptic connection weights between the neurons, *S*_1,2_ are the CPG inputs (i.e., load sensing feedback), and *o*_*i*_ are the CPG outputs. α is a sensory feedback connection (synaptic plasticity), automatically adjusted by dual rate learning (described in detail in the following section. See Equation 11). The CPG inputs are defined as:

(4) $$\begin{array}{l} S_{1}\left(t\right)=-F\left(t\right)cos\left(a_{1}\left(t\right)\right) \end{array}$$

(5) $$\begin{array}{l} S_{2}\left(t\right)=-F\left(t\right)sin\left(a_{2}\left(t\right)\right) \end{array}$$

where *F* is the negative continuous load sensing feedback at the leg. The sine and cosine functions of the neural activities *a*_1,2_ are used to derive a proper correlation between the neural activity and feedback. The functions are related to the phases of the CPG outputs^1^ *o*_1,2_ which differ by π/2 (see Figure 2). The strength of the sensory feedback connection can be adapted to regulate the amount of sensory feedback to the CPG-based control. Through this connection, the foot contact sensory feedback can reduce the leg speed when highly loaded at the end of the stance phase, while increasing the speed of the leg trajectory when it is unloaded at the end of the swing phase. This will allow the robot to adaptively adjust its leg movement to form a stable gait with good body weight distribution.

When implementing the neural control system on different robots, the proper value of α needs to be used for stable locomotion and this value might have to be changed in the face of unexpected situations, such as leg damage. The difficulty in determining an optimal value for all cases motivated us to develop an automatic process for continuously and dynamically adjusting the value without previous knowledge of the robot's morphology. The effect of the load sensing feedback on the CPG outputs is not the same for all CPGs or legs, but rather corresponds to the correlation of the feedback and the neural activities (Equations 4, 5). The variation of the influence on the CPGs will automatically yield phase differences among them, which will be translated into proper interlimb coordination (i.e., leg coordination). As a result, the robot will perform an adaptive gait. In this case, we do not have a fixed and predefined interlimb coordination, but rather a flexible one, since the gait obtained is derived from load sensing feedback, synaptic plasticity, neural activities, and body-environment interaction.

For intralimb coordination (i.e., joint coordination) in each leg of all tested robots, we project the CPG outputs to the TC-, CTr-, and FTi-joints indirectly through a CPG post processing unit. This post processing unit shapes the CPG outputs into the desired motor commands. In this case, a simple algorithm defining the swing and stance periods of the robots is applied (see Manoonpong et al., 2013). It translates the CPG outputs into ascending and descending slopes, finally controlling the joint movements in basic swing and stance phases. In our robot system here, we use position control. Thus, the motor commands correspond to the target TC-, CTr-, and FTi-joint positions.

### 2.2. Forward Model for Sensory Prediction

The learning algorithm implemented is inspired by Xiong et al. (2016), and takes advantage of an efference copy of the step movement, allowing comparison between expected or predicted sensor feedback for each leg position and actual sensory feedback. This difference can be used to tune the α value dynamically (Equations 1, 2) to adapt to changes in the sensory feedback.

**Figure 3:**
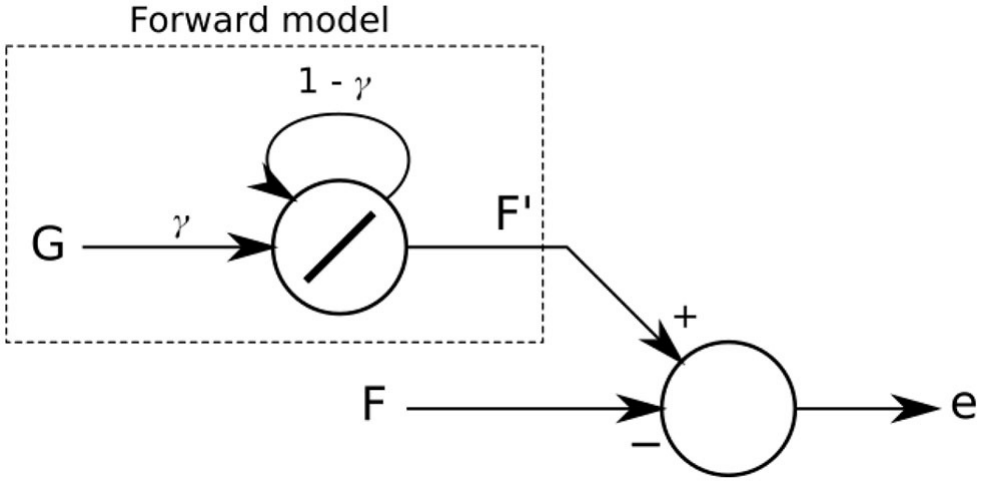
The forward model implemented as a linear recurrent neuron. Its output *F*′ is compared with the actual sensory feedback *F*. The difference of the comparison leads to an error *e* which is then used in the dual learning mechanism for sensory adaptation.

The forward model implemented (Figure 3) predicts that sensory feedback should be zero while the leg is lifted (swing phase) and a high value when the leg is on the ground (stand phase). In other words, a positive sensor value is expected when the leg touches the ground during the stance phase (downward position of the CTr-joint) and a zero value while the leg is in the air during the swing phase (upward position of the CTr-joint). This simple strategy tries to make the robot follow a stable stroke potentially giving the robot body good propulsion along the whole stance phase of the step. The forward model is given as:

(6) $$\begin{array}{l} F_{l}^{\prime }\left(t+1\right)=\gamma \cdot G_{l}\left(t\right)+\left(1-\gamma \right)\cdot F_{l}^{\prime }\left(t\right) \end{array}$$

(7) $$\begin{array}{l} G_{l}\left(t\right)=\{\begin{array}{cc} 0, & m_{0}^{l}\left(t+1\right)>m_{0}^{l}\left(t\right)\\ 1, & m_{0}^{l}\left(t+1\right)<=m_{0}^{l}\left(t\right) \end{array} \end{array}$$

where $F_{l}^{\prime }$ is the expected or predicted sensory value and γ is a factor in a range of [0, 1] defining the shape of the output signal of the forward model. In this study, we empirically adjust and set γ to 0.5. The signal $m_{0}^{l}$ is the motor command of the CTr-joint. When this motor value increases (moving the leg upward during swing phase) or decreases (moving the leg downward during stance phase), signal *G*_*l*_ is set to 0 or 1, respectively.

A forward model is used for each leg *l*. The difference between the forward model and its respective sensory input is calculated as:

(8) $$\begin{array}{l} e_{l}\left(t\right)=|F_{l}^{\prime }\left(t\right)-F_{l}\left(t\right)| \end{array}$$

**Figure 4:**
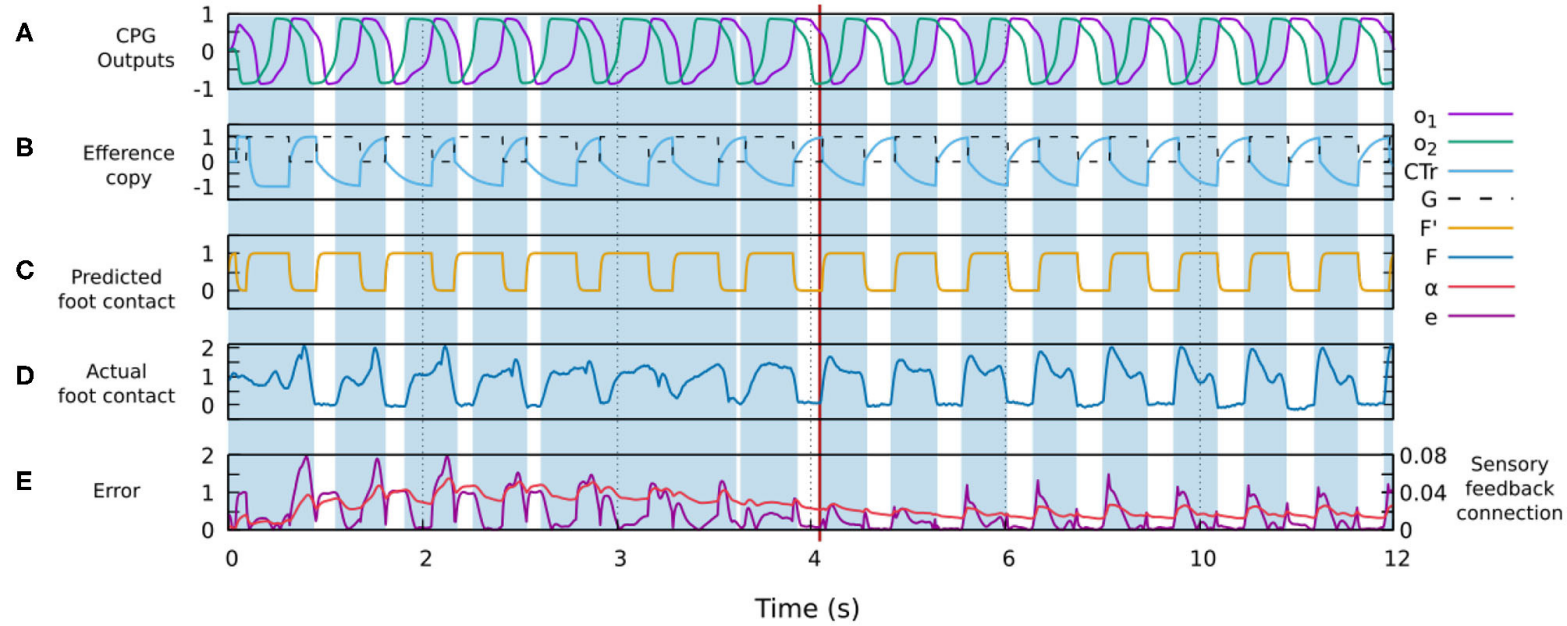
Changes in different signals of the control system during robot-environment interaction and sensory adaptation to form a gait. **(A)** The outputs of the CPG for driving a leg (*o*_1_, *o*_2_). **(B)** The original efference copy (motor command of the CTr-joint) and the transformed one *G* (i.e., input of the forward model, Figure 3). **(C)** The predicted foot contact sensor signal *F*′ as the output of the forward model (Figure 3). **(D)** The actual foot contact sensory signal *F*. **(E)** The error signal *e* and sensory feedback connection α. All these signals were recorded from the simulation of the four-legged robot. Areas colored blue show the stance phases while the white areas refer to the swing phases. The red line indicates the point at which the robot starts showing a stable gait. In this case, a stable gait is quickly formed within about 6 s.

Figure 4 shows the signals involved in the forward model. One can observe how the actual sensor value changes providing a better match with the predicted signal generated by the forward model. The change is due to the robot-environment interaction and sensory adaptation, described by the shadowed areas representing the stance phases of the foot contact sensory feedback.

### 2.3. Dual Rate Learning for Sensory Adaptation

In order to reduce the error produced by the mismatch between predicted and actual sensory feedback signals, the strength of the sensory feedback on the CPG (i.e., α of Equations 1, 2) is continuously adjusted online at each leg using the dual rate learning process (Smith et al., 2006). The implemented controller combines fast and slow adaptation processes (i.e., fast and slow learners) arranged in parallel. The fast adaptation performs rapid initial learning. However, it forgets quickly while the slow adaptation contributes to long-term retention, but it adapts slowly. The use of the two parallel adaptations at different time scales leads to the fast and stable convergence of sensory feedback strength.

**Figure 5:**
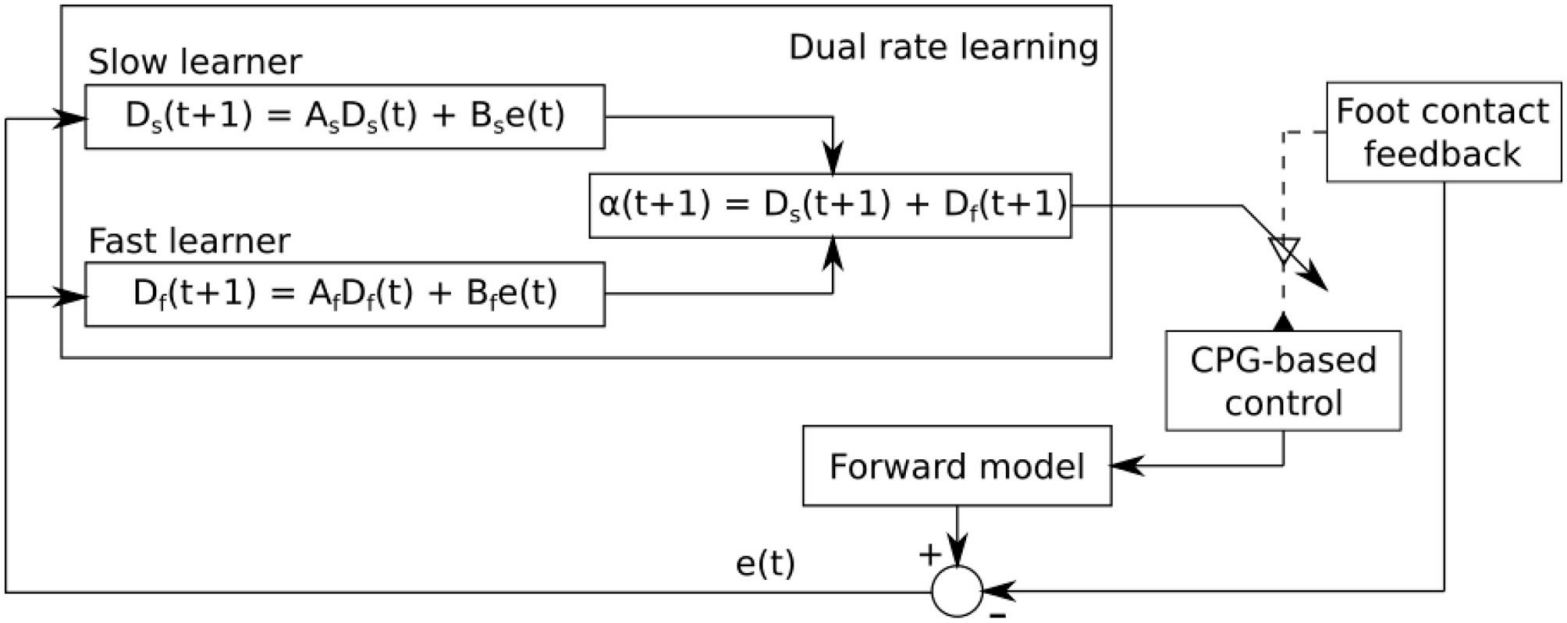
Dual rate learning for the sensory feedback strength adaptation of CPG-based control. The sensory information from the foot contact sensor at each leg is transmitted to its corresponding CPG-based control. It shapes the CPG outputs to obtain a proper phase between legs. The learning process continuously adapts the strength of the sensory feedback to ensure that proper sensory information is transmitted to its corresponding CPG-based control. The forward model receives an efference copy (i.e., CTr motor command) and translates it into predicted foot contact sensory feedback which is then compared to the actual foot contact sensory feedback. The difference between them is sent to the learning process for sensory feedback strength adaptation.

**Figure 6:**
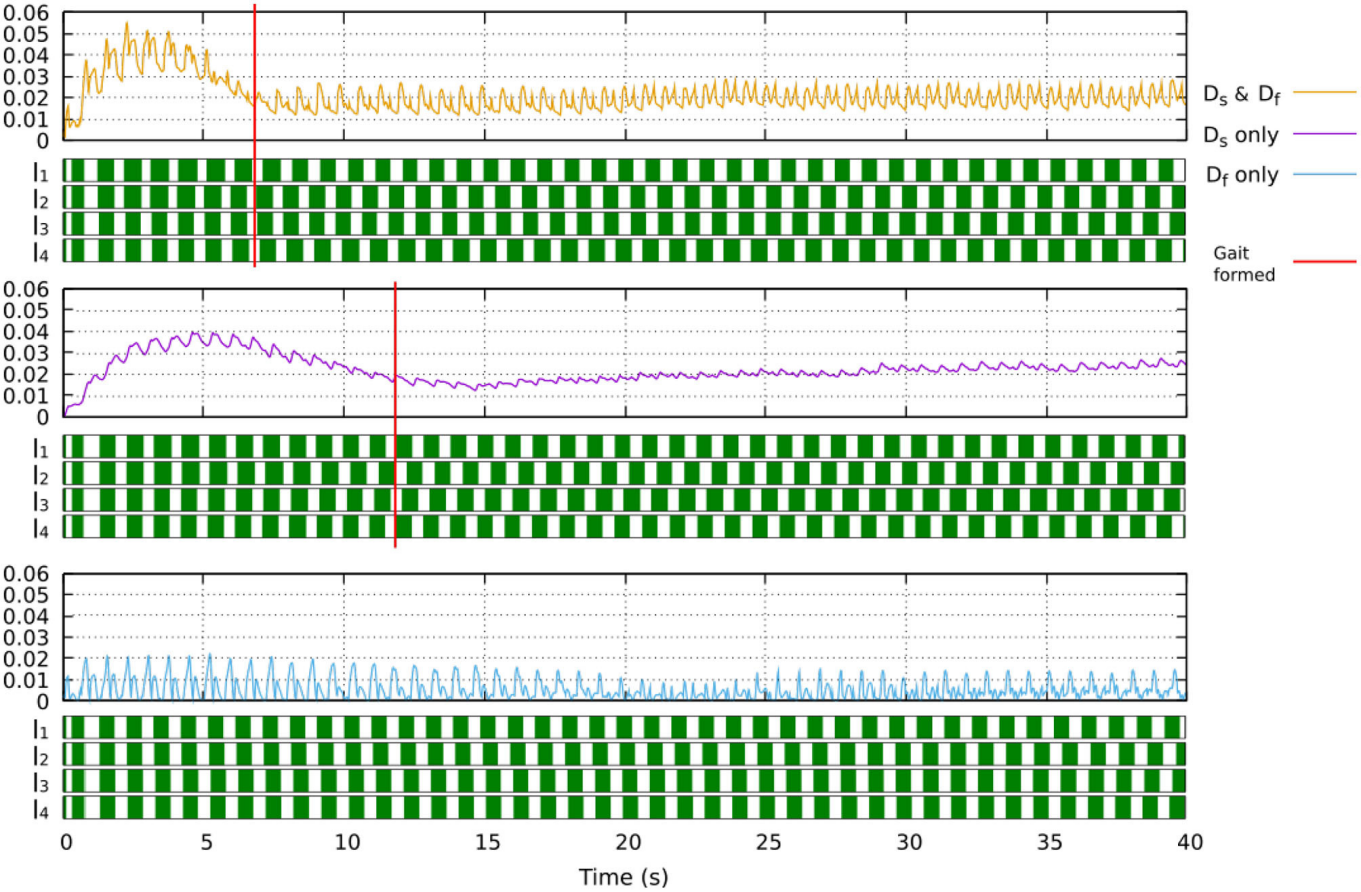
The effect of the learning process when only slow learner, only fast learner, or their combination (dual learner) is used. Using the dual learner leads to fast convergence where a stable gait (showing clear swing and stance phases) is formed at around 7 s. Using only slow learner leads to slow convergence where a walking gait is formed at around 12 s, although this gait is more irregular and unstable. Using only fast learner leads to instability where a stable gait cannot be formed. A video showing robot locomotion with different learners can be seen at: www.manoonpong.com/Frontiers2020/DifferentLearning.mp4 (or see Video S9).

In this way, a proper feedback strength can be obtained after a few walking steps. Here, each adaptation process receives the same error and adapts the sensory feedback strength accordingly, as shown in the following equations:

(9) $$\begin{array}{l} D_{f}\left(t+1\right)=A_{f}D_{f}\left(t\right)+B_{f}e\left(t\right) \end{array}$$

(10) $$\begin{array}{l} D_{s}\left(t+1\right)=A_{s}D_{s}\left(t\right)+B_{s}e\left(t\right) \end{array}$$

(11) $$\begin{array}{l} \alpha \left(t+1\right)=D_{f}\left(t+1\right)+D_{s}\left(t+1\right) \end{array}$$

where *D*_*f*_ is the output of the fast adaptation. *D*_*s*_ is the output of the slow adaptation. α is the combination of the two outputs (i.e., sensory feedback strength adaptation). *e* is the error calculated from the difference between the predicted and actual sensory feedback (see Equation 8). *B*_*s*_ and *B*_*f*_ are the learning rates of the slow and fast adaptation mechanisms, respectively. *A*_*s*_ and *A*_*f*_ are retention factors of the slow and fast adaptations, respectively. The parameters are determined as *A*_*s*_ > *A*_*f*_ and *B*_*s*_ < *B*_*f*_. The fast adaptation mechanism thus adapts more rapidly as indicated by a higher learning rate but also forgets more rapidly as indicated by a lower retention factor. In this study, we set *A*_*s*_ = 0.992, *A*_*f*_ = 0.57, *B*_*s*_ = 0.0004, *B*_*f*_ = 0.005. These values were used in Xiong et al. (2016). Figure 5 shows the implementation of the dual rate learning process. In this learning process, using only slow adaptation will lead to slow converge while using only fast adaptation will lead to instability (see Figure 6).

## 3. Experimental Setup

To validate the performance of our proposed neural control system with sensory adaptation, we simulated different robots with 4, 6, 8, and 20 legs. We used the robot simulation framework LpzRobots (Martius et al., 2010) based on the Open Dynamics Engine (Smith, 2006). Each robot has the same body and leg structures (see Figure 1). Each body segment consists of one or two pairs of legs. Each leg has three joints (see Figure 1) inspired by those of invertebrates. We used a universal passive joint with limited rotation to connect between body segments. This allows for small passive body movements and dynamics for stability.

**Figure 7:**
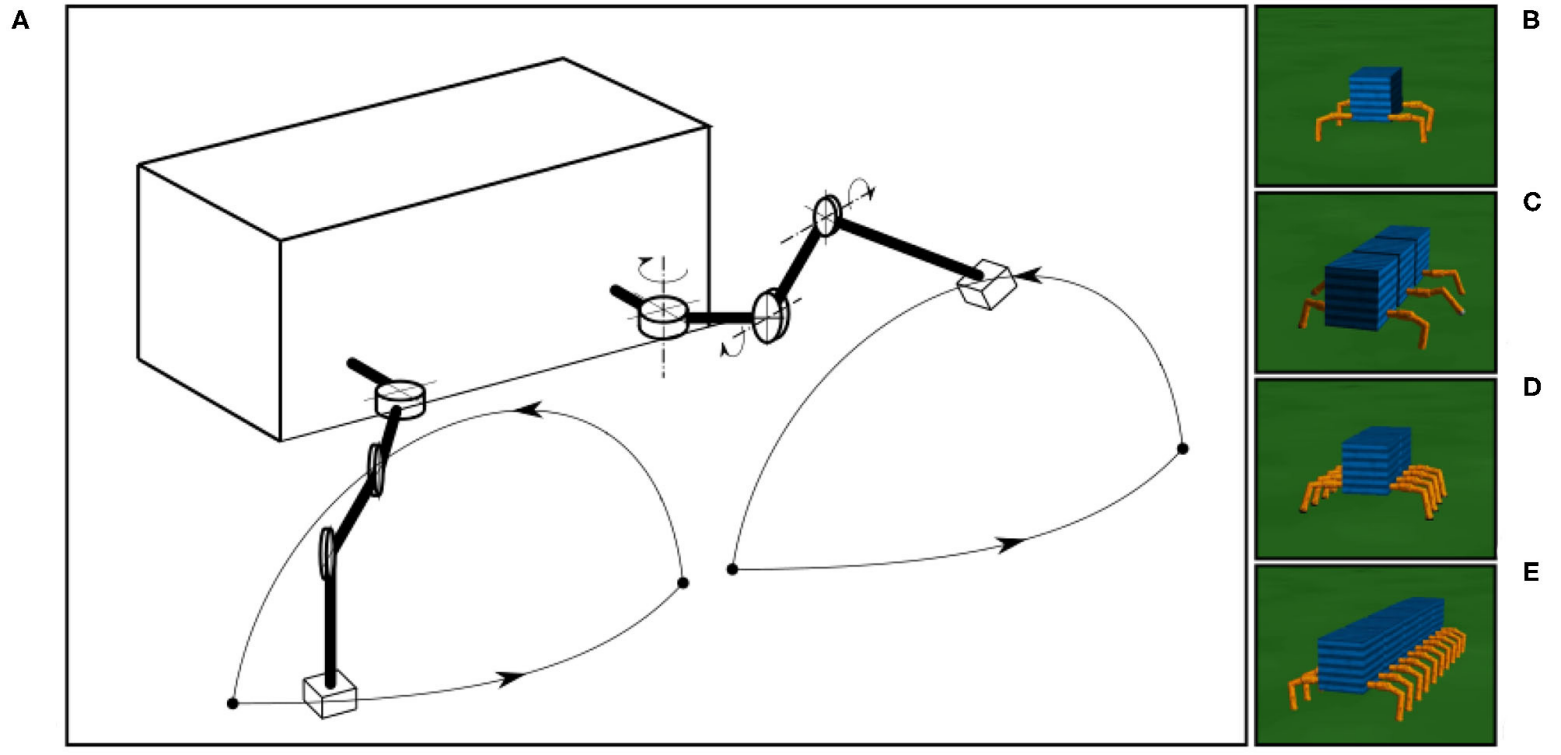
The simulated robots and leg trajectory of a robot in one step. **(A)** The trajectory consists of stance and swing phases. The stance phase follows an almost straight trajectory where the TC-joint moves backward and CTr- and FTi-joints are set to certain positions. The swing phase forms an arch with a movement combination of the three joints (i.e., TC-joint moves forward, CTr-joint moves up, and FTi-joint extends). The simple leg movement is implemented for walking on a flat surface only. Walking on rough terrain (which is not the focus of this study), requires additional control mechanisms, such as reflexes (Manoonpong et al., 2013). **(B–E)** The four-, six-, eight-, and 20-legged robots used in this study.

All simulated 4-, 6-, 8-, and 20-legged robots have similar leg movements with the same amplitude, allowing the observation of different walking patterns or gaits with respect to different numbers of legs. In this study, we focus only on an adaptive process for the interlimb coordination or the coordination between legs while the intralimb coordination or the coordination between joints within the leg are predefined. The leg trajectory resulting from the predefined intralimb coordination is shown in Figure 7A.

The tests for different robots were performed on a flat terrain (Figures 7B–E). The leg trajectory, all environment variables (e.g., gravity, friction), and sensory noise had the same settings for all robot experiments. We added a Gaussian-distributed noise with a standard deviation of 10%. In all experiments, we initiated the robots with an irregular gait on a flat terrain, where all legs moved in phases with a frequency of 1.4 Hz. The sensory feedback strength α was also initialized to zero, i.e., no connection between the sensory feedback and CPG-based control. As previously mentioned, in this distributed neural control approach, no CPG-based control module has any connection or direct communication with another. The gaits will emerge from the body-environment interaction, i.e., physical communication (Owaki et al., 2012) through foot contact sensory feedback and the proposed sensory adaptation in this study.

## 4. Results

In this section, we present the results of the experiments with simulated robots (Figure 7). The performance of the proposed neural control system was evaluated in two scenarios. The first of which accessed the self-organized locomotion of the robots using different morphologies. The second was conducted to show the adaptability of the control approach in dealing with the amputation of different legs. The leg amputation was simply performed by lifting it above the ground or keeping it fixed in a certain position. In order to statistically evaluate the control performance, each robot was tested 200 times in the first scenario and 500 times in the second. During the experiments, we measured the average walking speed once the robots had moved forward.

**Figure 8:**
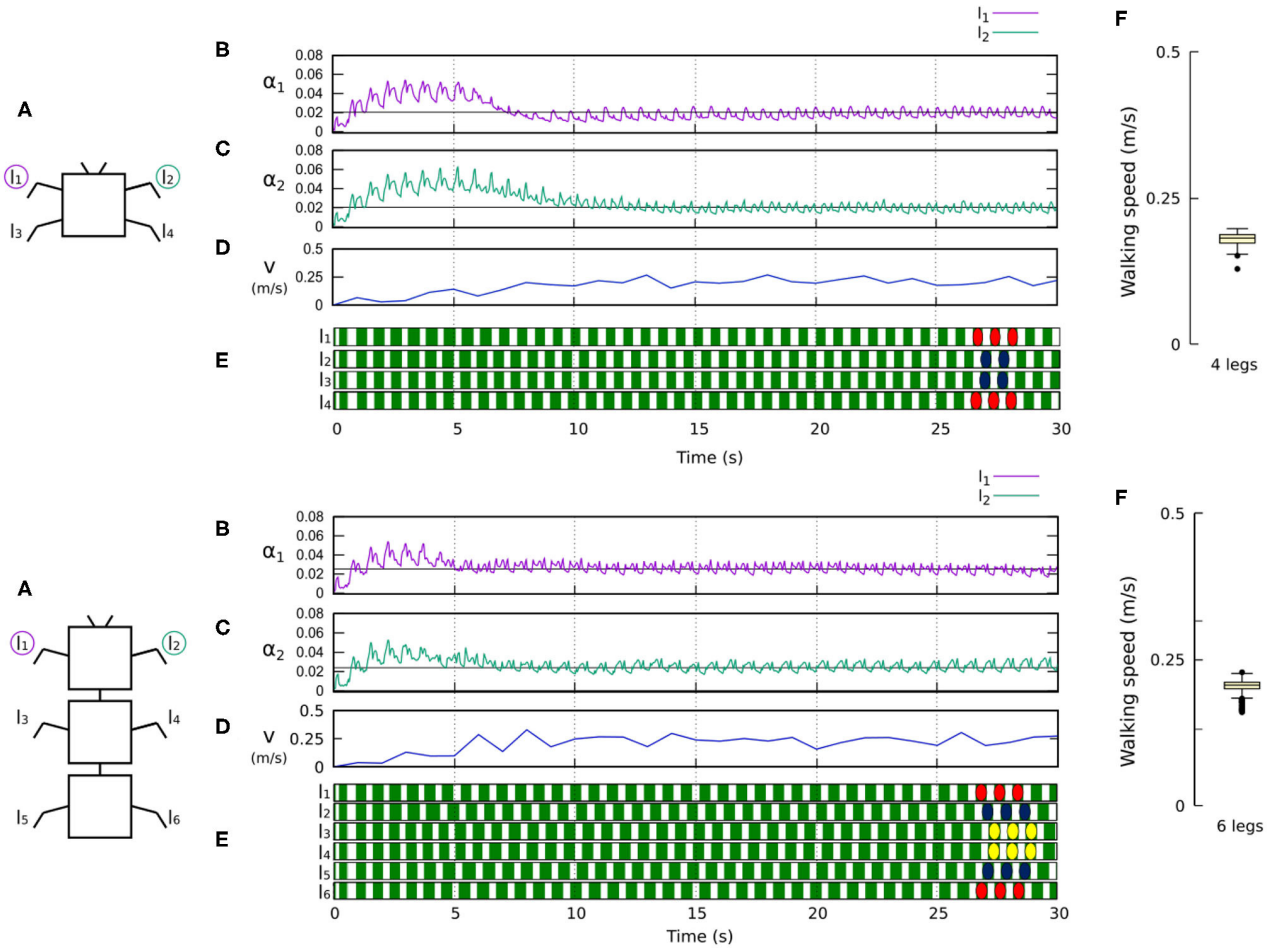
Results for the four- and six-legged robots. For each configuration: **(A)** Scheme of the tested robot. **(B,C)** Sensory feedback strengths during simulation on the left front leg (*l*_1_) and right front leg (*l*_2_), respectively. A black line indicates the average value to which each strength converges. **(D)** The change in the robot walking speed during the simulation. **(E)** The swing and stance phases of each leg of the robot, defined by motor commands. Green areas represent stance phases, while white areas correspond to swing phases. Colored marks are used to visualize the formed gaits, namely a trot gait for four legs and a bipod gait for six legs. **(F)** Average walking speed from 200 tests. Videos showing examples of self-organized locomotion of the four- and six-legged robots can be viewed at: www.manoonpong.com/Frontiers2020/4legs.mp4 (or see Video S1) and www.manoonpong.com/Frontiers2020/6legs.mp4 (or see Video S2), respectively. Note that all sensory feedback strengths of the four- and six-legged robots are shown in Figures S1, S2.

**Figure 9:**
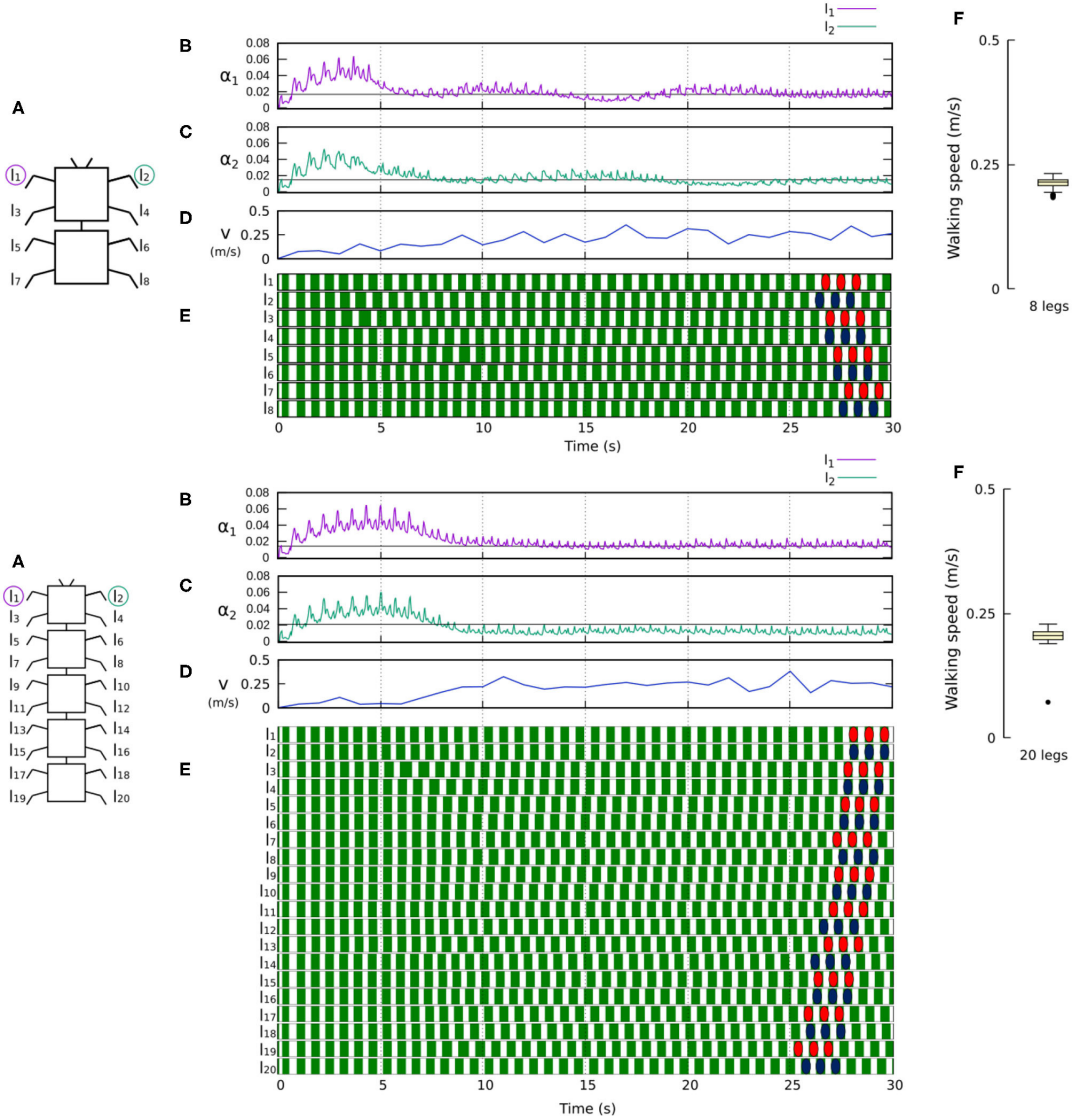
Results for the 8- and 20-legged robots. For each configuration: **(A)** Scheme of the tested robot. **(B,C)** Sensory feedback strengths during simulation on the left front leg (*l*_1_) and right front leg (*l*_2_), respectively. A black line indicates the average value to which each strength converges. **(D)** The change in the robot walking speed during the simulation. **(E)** The swing and stance phases for each leg of the robot, defined by motor commands. Green areas represent stance phases, while white areas correspond to swing phases. Colored marks are used to visualize the formed gaits of the 8- and 20-legged robots. In this case, both robots show metachronal-like gaits. **(F)** Average walking speed from 200 tests. Videos showing examples of self-organized locomotion in the 8- and 20-legged robots can be viewed at: www.manoonpong.com/Frontiers2020/8legs.mp4 (or see Video S3) and www.manoonpong.com/Frontiers2020/20legs.mp4 (or see Video S4), respectively. Note that all sensory feedback strengths of the 8- and 20-legged robots are shown in Figures S3, S4.

Figures 8, 9 show that the control approach was able to quickly adapt the sensory feedback strength and generate self-organized locomotion within 5–10 s. The gaits emerging from the 4, 6, 8, and 20-legs were similar to those observed in animals; i.e., a trot gait in the four-legged robot, a bipod gait in the six-legged robot^2^ (Ramdya et al., 2017), and metachronal wave gaits in the 8- and 20-legged robots^3^ (Bowerman, 1975; Spagna and Peattie, 2012; Kano et al., 2017a; Yasui et al., 2017). The average walking speed of all robots was ≈ 0.2 m/s.

**Figure 10:**
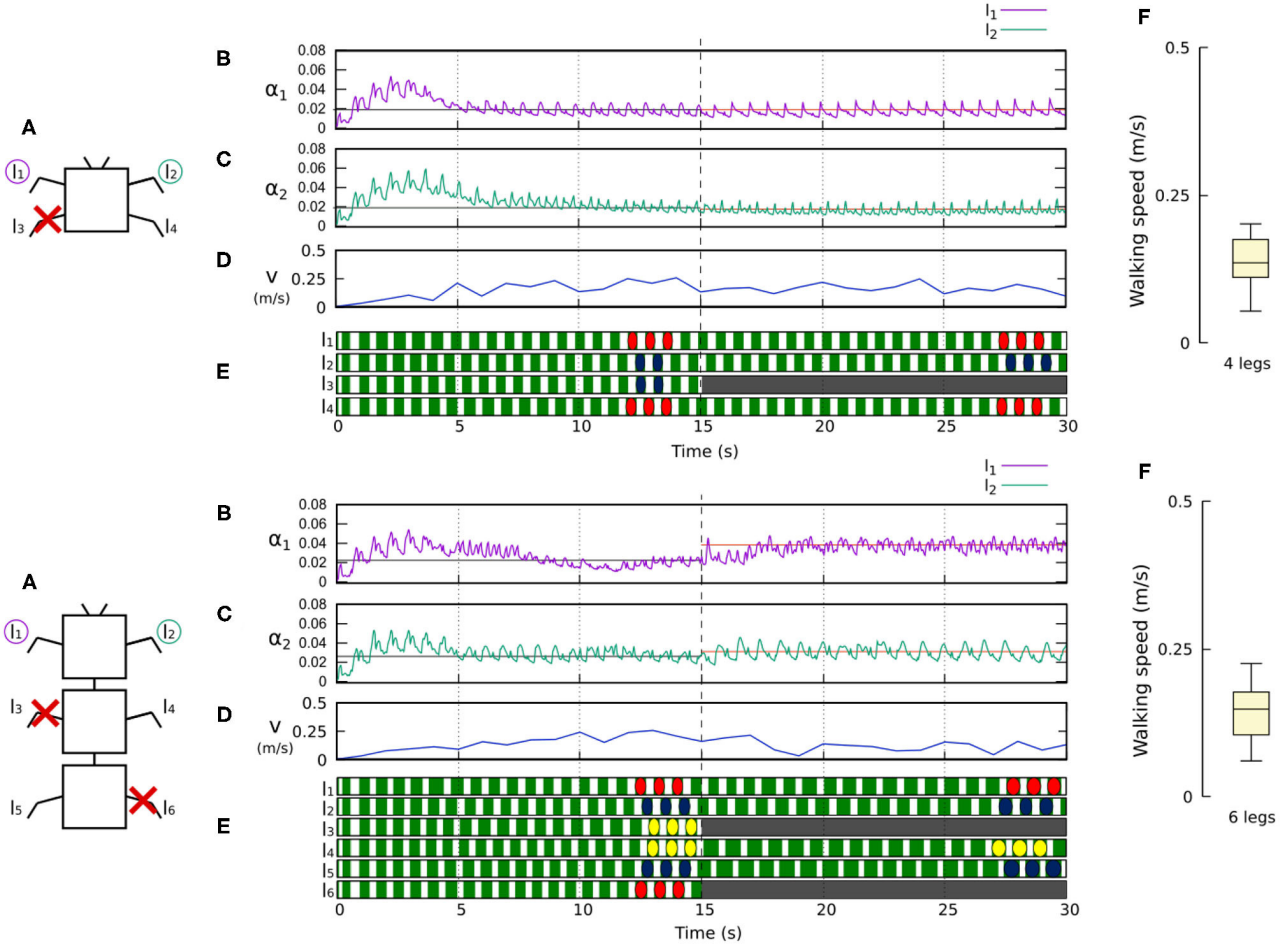
Results for the four- and six-legged robots when disabling certain limbs after 15 s of simulation. For each configuration: **(A)** Scheme of the tested robot, including the limbs amputated (red cross) in this experiment. **(B,C)** Sensory feedback strengths during simulation on the left front leg (*l*_1_) and right front leg (*l*_2_), respectively. A line indicates the average value to which each strength converges. The convergence values before and after the amputation are drawn in black and red, respectively. **(D)** The change in the robot walking speed during the simulation. **(E)** The swing and stance phases of each leg of the robot, defined by motor commands. Green areas represent stance phases, while white areas correspond to swing phases. After amputation occurs, the state of the disabled limbs is represented in gray. Colored marks are used to visualize the formed gaits. **(F)** Average walking speed from 500 tests. For each simulation, a random number of arbitrary limbs was disabled, investigating the ability of the system to adapt to different morphologies. Videos showing examples of adaptation to leg damage of the four- and six-legged robots can be viewed at: www.manoonpong.com/Frontiers2020/4legsDamage.mp4 (or see Video S5) and www.manoonpong.com/Frontiers2020/6legsDamage.mp4 (or see Video S6), respectively. Note that all sensory feedback strengths of the four- and six-legged robots with leg damage are shown in Figures S5, S6.

**Figure 11:**
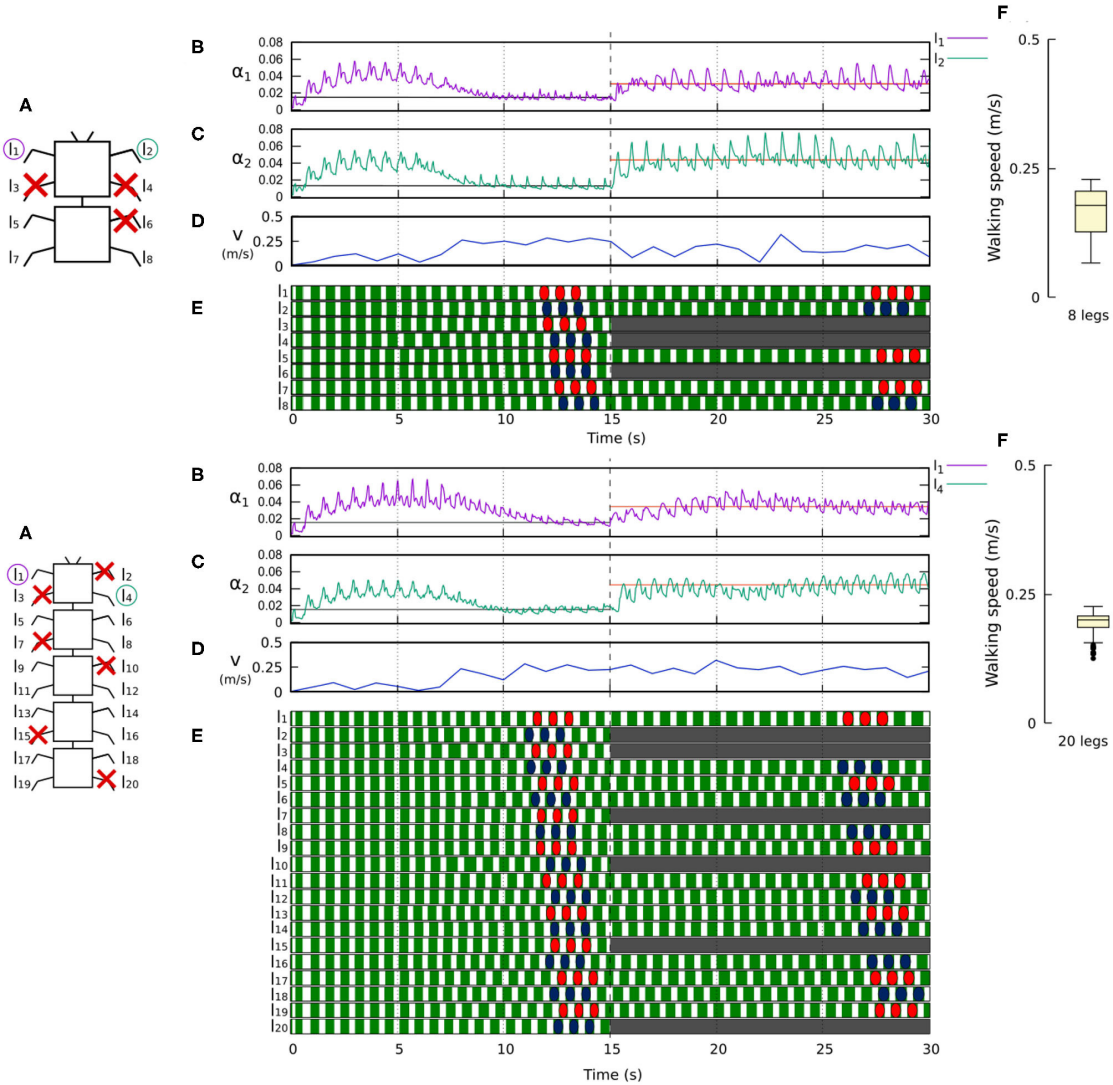
Results for the 8- and 20-legged robots when disabling certain limbs after 15 s of simulation. For each configuration: **(A)** Scheme of the tested robot, including the limbs amputated (red cross) in this experiment. **(B,C)** Sensory feedback strengths during simulation on the left front leg (*l*_1_ in both 8- and 20-legged robots) and a right leg (*l*_2_ in the eight-legged robot and *l*_4_ in the 20-legged robot), respectively. A line indicates the average value to which each strength converges. The convergence values before and after the amputation are drawn in black and red, respectively. **(D)** The change in the robot walking speed during the simulation. **(E)** The swing and stance phases of each leg of the robot, defined by motor commands. Green areas represent stance phases, while white areas correspond to swing phases. After amputation occurs, the state of the disabled limbs is represented in gray. Colored marks are used to visualize the formed gaits. **(F)** Average walking speed from 500 tests. For each simulation, a random number of arbitrary limbs was disabled, investigating the ability of the system to adapt to different morphologies. Videos showing examples of adaptation to leg damage of the 8- and 20-legged robots can be viewed at: www.manoonpong.com/Frontiers2020/8legsDamage.mp4 (or see Video S7) and www.manoonpong.com/Frontiers2020/20legsDamage.mp4 (or see Video S8), respectively. Note that all sensory feedback strengths of the 8- and 20-legged robots with leg damage are shown in Figures S7, S8.

Figures 10, 11 demonstrate that the control approach was able to quickly adapt the sensory feedback strength to new values within a few seconds of amputation and new gaits emerged to allow the robots to locomote. The average walking speed of all robots was ≈0.125–0.175 m/s. In this case, the statistical study on controller adaptability was conducted using multiple tests with a randomized number of amputations of arbitrary legs on the robots. Since some morphologies did not show any gait when amputating too many legs, the maximum number of amputations was set to half of the total legs for each robot morphology; i.e., two for the four-legged robot, three for the six-legged robot, four for the eight-legged robot, and 10 for the 20-legged robot.

After running multiple simulations with different amputation configurations for each robot, the results show that in most cases the control approach was able to find gaits, allowing the robots to move forward. The variance of the results is higher than in the first experiment due to the large number of configurations formed by the amputations. For some configurations, the robots could manage to move effectively while others made it more difficult for the robots to move forward.

By determining the adaptation of the robots to the amputations, in Figures 10, 11, one can see how the dual rate learning mechanism adapts the feedback strength of the different legs (e.g., left and right front legs) to deal with unexpected new body conditions. It is also possible to observe how the robot gaits change to compensate for the lack of proper support resulting from the leg amputation.

**Figure 12:**
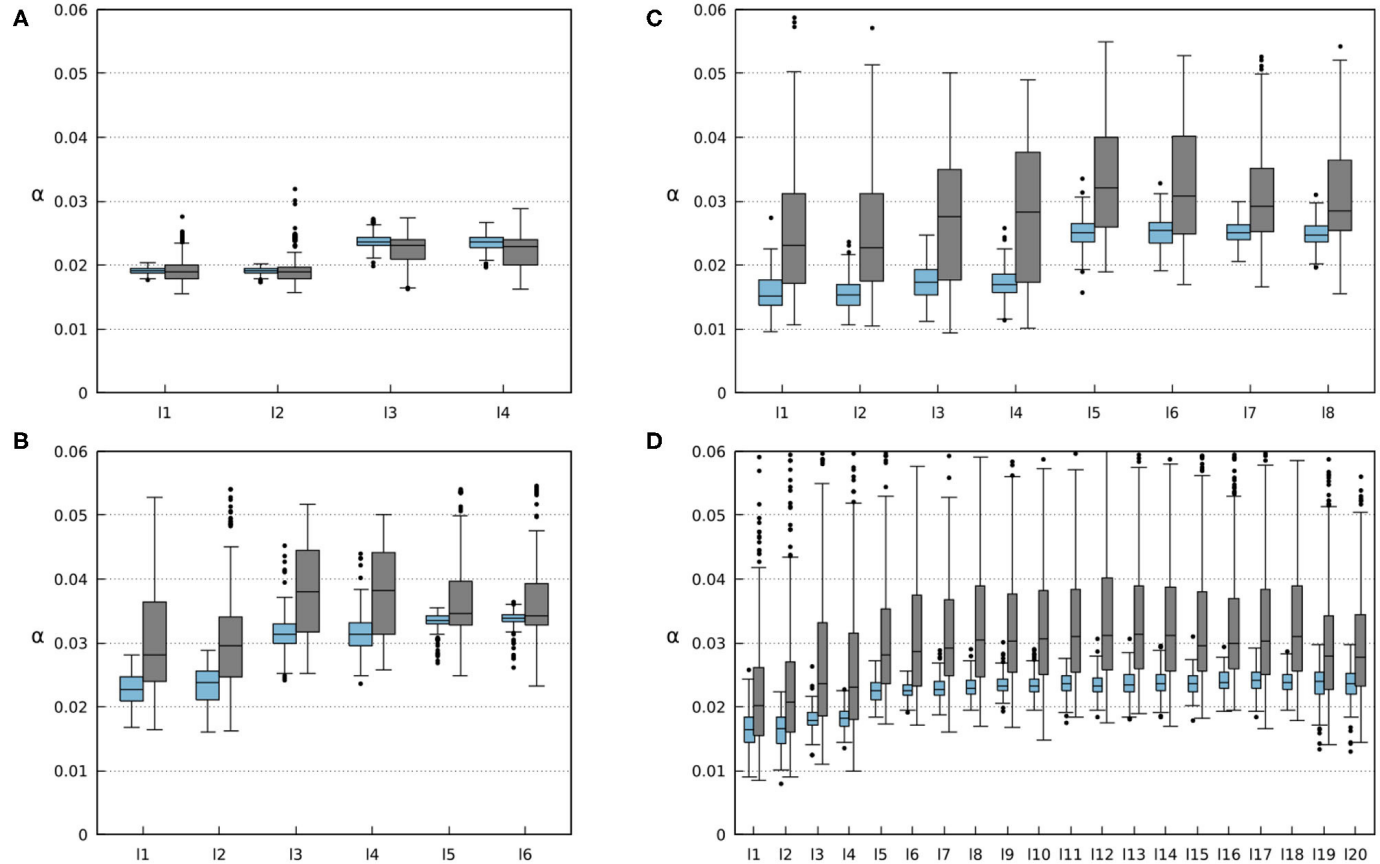
Average weights of the foot contact sensory feedback (i.e., sensory synaptic strength). Each *li* box represents the results for each *i* leg of the robots. Blue boxes represent the results for 200 runs on robots without any amputations. Gray boxes represent the results for 500 runs where randomized amputations were performed to obtain different configurations. In this case, the robots were amputated from the beginning of the simulation where the robots were still able to form gaits. **(A)** Results for the four-legged robot. **(B)** Results for the six-legged robot. **(C)** Results for the eight-legged robot. **(D)** Results for the 20-legged robot.

The ability of the robots to find a stable gait depends on the convergence of sensory feedback strength (i.e., synaptic weight). The convergent weights during the experiments are shown in Figure 12. One can observe a range of convergence for each leg of every robot with and without amputations. In simulations where no limbs were amputated, this range is quite narrow, and the robot tends to keep a relatively constant weight. However, the amputation experiments show how the changes in the robot morphology can affect the weights needed to obtain a stable gait. By analyzing the results shown in Figure 12, Figures S5–S8, one can observe that the weights of remaining legs increase after amputation. This is due to the new weight distribution across the remaining legs. Increasing the weights leads to stronger inhibition to the CPGs (see Equations 1, 2, 4, 5), resulting in a longer stance phase. This way, the robot can readjust its gait to obtain a new stable one.

## 5. Discussion and Conclusion

The previous sections present a self-organized locomotion control system with the ability to quickly adapt to changes in the robot morphology. In this section, we discuss the advantages of this mechanism, comparing the proposed neural control system with other solutions and describing some of the remaining issues for improving this approach.

Previous research (Owaki et al., 2012, 2017; Barikhan et al., 2014; Aoi et al., 2017; Kano et al., 2017b, 2019; Ambe et al., 2018) has shown the possibility of offloading the task of interlimb coordination toward body-environment interactions and adaptation to physical damage. By using sensory feedback to trigger reactions in local (decentralized) CPGs and drive individual leg movements, this approach is able to form walking patterns in robots without direct control over the interlimb coordination process. The approach is supported by experimental results showing that invertebrate locomotion (e.g., insects, millipedes, centipedes) is controlled in a similar way, using decentralized CPGs for individual limbs (Bässler and Büschges, 1998; Kano et al., 2017a; Yasui et al., 2017, 2019). The biological study suggests that walking behavior in invertebrates relies highly on decentralized mechanisms and load sensing feedback.

The use of decentralized CPGs is extremely advantageous for walking robots, as it does not require the costly computation of inverse kinematics or a precise kinematic model. While it is possible to generate locomotion under fixed control parameters (e.g., sensory feedback strength), the parameters need to be empirically adjusted. The addition of learning mechanisms for adjusting control parameters online leads to flexibility and adaptability, and ultimately to more robust and general control. Compared to advanced machine learning techniques [like, the Intelligent Trial and Error algorithm (IT&E, Cully et al., 2015) and a greedy random-mutation hill climber algorithm with self-modeling (GRSM, Bongard et al., 2006)] for locomotion generation and adaptation to damage, our proposed mechanism shows advantages over the techniques in the following aspects:

- It can quickly and continuously adapt to structural changes in the robot (e.g., leg damage and different configurations) online within a few walking steps (i.e., 5–10 s) and without the need of pretrained behavior-performance map (as shown in IT&E^4^) or internal morphological models (as shown in GRSM^5^).
- It does not need a complex trial-and-error or optimization process for locomotion generation and adaptation (as needed in IT&E and GRSM); instead, it simply exploits body-environment interaction with sensory adaptation based on online dual rate learning and synaptic plasticity.
- It is computationally less expensive than IT&E and GRSM.
- It does not need additional exteroceptive feedback (like RGB-D camera as used in IT&E) or global position feedback (as used in GRSM) to measure or evaluate robot performance; instead, it only relies on foot contact or load sensing feedback.

Furthermore, due to the complex processes of the machine learning techniques, they will become much more problematic when applying to a robot with a large number of degrees of freedom, such as the 20-legged robot, which includes 65 moving parts and 60 active joints, tested in this study.

The method proposed here is based on the existing body-environment interaction approach for interlimb coordination to which we have now contributed by introducing online learning. The learning, which incorporates forward models, automatically adapts sensory synaptic plasticity (i.e., the sensory feedback strength parameter) on the CPG network. This new mechanism adds a second timescale adaptation to the system. To fully understand the dynamics of the system, it is necessary to differentiate between the two timescale adaptations: the gait formation process (fast time scale adaptation, 0–5 s in Figures 8, 9) and the tuning of synaptic strength in sensory feedback (slow time scale adaptation, after 5 s in Figures 8, 9). By considering the amputation experiments, one can see two types of sensory plasticity (Pyza, 2013): One is so-called experience-induced plasticity and the other lesion-induced plasticity. The experience-induced plasticity is driven by stimulation (Bozorgmehr et al., 2013). In our case here, it is used to form a gait as can be observed in the first period; 0–15 s in Figures 10, 11. The lesion-induced plasticity occurs after injury (Pfister et al., 2013) and, in our case here, when the robots were amputated during the second period after 15 s, as shown in Figures 10, 11. Such plasticity has been observed in invertebrate sensory systems during development and in the adult stage (Bozorgmehr et al., 2013; Lakes-Harlan, 2013; Pfister et al., 2013; Pflüger and Wolf, 2013).

In our control approach, walking patterns emerge from the reaction of each leg to sensory information. Consequently, the gait formation and sensory plasticity mechanisms need to work simultaneously. On the one hand, the robot cannot form a gait unless the sensory feedback synaptic strength is strong enough. On the other, the adjustment of sensory feedback synaptic strength depends on the formation of a gait to converge. As a result, the learning process of each leg is affected by the movement and dynamics of the robot. The process exploits the actual sensory feedback and the prediction provided by the forward model to adjust the sensory feedback synaptic strength, which is increased when the actual feedback and its expectation do not match. If the robot is not able to form a stable gait, the mismatch or error will keep increasing the synaptic strength. By increasing the strength, the sensory feedback will influence the CPG network and thereby shape the CPG outputs (see Equations 1, 2 and Figure 4). Since the process occurs in parallel in each CPG network of every leg, proper phases between the CPG networks will be finally obtained. In analogy to biological systems, the dual rate learning system can be considered as a serotonergic system or an extrinsic modulator that releases serotonin to modulate or influence sensory synaptic plasticity (i.e., sensory gain) in invertebrate nerve cord (Klein et al., 1982; van Haeften et al., 1993; Majeed et al., 2016; Le Gal et al., 2017; Upreti et al., 2019) or vertebrate spinal cord (Stutzmann et al., 1998; Deemyad et al., 2013; Lottem et al., 2016; Avery and Krichmar, 2017) while the sensory feedback projecting to the CPG network can be determined as an extrinsic modulatory input which alters the network dynamics (Katz, 1998; Morgan et al., 2000; Marder, 2012).

The sensory plasticity or adaptation provides multiple advantages compared to the previous model which has no sensory plasticity (Aoi et al., 2007; Owaki et al., 2012; Ambe et al., 2013; Barikhan et al., 2014). It provides an automatic process to tune or find proper sensory feedback contribution to obtain self-organized locomotion. It also introduces the dynamics of sensory feedback gain which leads to fast adaptation (see Figure S9); implicitly indicating an ability to adapt to changes in robot morphology and weight distribution. Furthermore, simulation results show that our approach allows robots with different morphologies to form walking gaits similar to insects. Recent work on sensory adaptation has been also proposed by Ishige et al. (2019). There, they used a combination of CPG-based control and an episode-based reinforcement learning (RL) method (i.e., policy gradients with parameter-based exploration) which was applied to caterpillar-like soft robots. The RL method was used to optimize mechanosensory feedback (sensory adaptation) to the CPG-based control, which controls actuators in the robot. This method, while impressive in their own right, still requires high computational effort (approx. 100–400 epochs^6^) to optimize the feedback to the CPG-based control for generating effective robot crawling behavior.

Taken together, this study proposes general locomotion control for multiple robot configurations and injury compensation. This control system is based on decentralized CPGs with load sensing feedback, a forward model for sensory prediction, and online learning for continuous sensory adaptation. These three components, interacting with body dynamics, can autonomously form robot gaits and compensate for leg damage without the manual tuning of sensory feedback strength required by the previous studies (mentioned above). When applied to various legged robots, different animal-like gaits can be observed, including a typical trot gait for four legs, a fly-like bipod gait for six legs (Ramdya et al., 2017), a scorpion-like metachronal wave gait for eight legs (Bowerman, 1975; Spagna and Peattie, 2012), and a millipede or centipede-like metachronal wave gait for 20 legs (Kano et al., 2017a; Yasui et al., 2017). While this approach shows effective results, it still has a limitation. So far, posture control has not been integrated into the control system. Therefore, robots with four and six legs can become unstable if their legs are amputated since parts of the body may drop on the ground. Furthermore, the robot can only deal with walking on a flat terrain. Walking on uneven or complex terrains will require additional control mechanisms, like local leg extension, elevation control (Manoonpong et al., 2013), and impedance control with online adaptation (Xiong and Manoonpong, 2018; Sun et al., 2020). Thus, in the future, we will further investigate the integration of posture control, local leg control, and muscle models into the control system to achieve self-organized locomotion with high adaptability for traversing on complex terrains.

## Data Availability Statement

All datasets generated for this study are included in the article/Supplementary Material.

## Author Contributions

PM provided the general direction of the project, supervised the development of the neural control system, and helped with data analysis. AM-B developed the neural control system, performed the robot experiments, and analyzed the data. The manuscript was written by AM-B and PM. Both authors contributed to the article and approved the submitted version.

## Conflict of Interest

The authors declare that the research was conducted in the absence of any commercial or financial relationships that could be construed as a potential conflict of interest.

## Footnotes

^1^The CPG outputs represent the oscillator phases in the Tegotae-based approach (Owaki et al., 2017).

^2^This gait is found in Drosophila. It is similar to the vertebrate running trot and faster than a typical tripod gait. In this gait, each front leg moves almost at the same time as the opposite hind leg, while the middle legs move together.

^3^In scorpions, the gait shows metachronal alternating tetrapods with a 10% lag between legs within a tetrapod. In millipedes/centipedes, the gait exhibits propagating density waves running from the hind legs to the front legs.

^4^IT&E starts with random controllers and then performs stochastic, population-based, optimization. This process requires 20 million iterations for creating one behavior map (containing over 13,000 high-performing behaviors or gaits), which lasted roughly 2 weeks on one multi-core computer.

^5^The algorithm is based on an evolutionary process where new morphological models are produced by copying the originals, introducing small random modifications, and reevaluating them. This process uses the mean Euclidean distance between the centroid of each model body part, and where the centroid should be for performance evaluation. The process requires 200 iterations to obtain the best models.

^6^One epoch consists of 20 independent episodes and one episode lasts 50 s. Thus, the process takes several hours up to a day.

## Supplementary Material

Figure S1 Adaptation of the sensory feedback connection of each limb (α_*i*_) of the four-legged robot during 30 s of simulation. A video showing an example of self-organized locomotion of the four-legged robot can be viewed at: www.manoonpong.com/Frontiers2020/4legs.mp4.

Figure S2 Adaptation of the sensory feedback connection of each limb (α_*i*_) of the six-legged robot during 30 s of simulation. A video showing an example of self-organized locomotion of the six-legged robot can be viewed at: www.manoonpong.com/Frontiers2020/6legs.mp4.

Figure S3 Adaptation of the sensory feedback connection of each limb (α_*i*_) of the eight-legged robot during 30 s of the simulation. A video showing an example of self-organized locomotion of the eight-legged robot can be viewed at: www.manoonpong.com/Frontiers2020/8legs.mp4.

Figure S4 Adaptation of the sensory feedback connection of each limb (α_*i*_) of the twenty-legged robot during 30 s of simulation. A video showing an example of self-organized locomotion of the twenty-legged robot can be viewed at: www.manoonpong.com/Frontiers2020/20legs.mp4.

Figure S5 Adaptation of the sensory feedback connection of each limb (α_*i*_) of the four-legged robot during 30 s of simulation, where one limb was disabled after 15 s. A video showing an example of self-organized locomotion of the four-legged robot can be viewed at: www.manoonpong.com/Frontiers2020/4legsDamage.mp4. Note that the sensory feedback connections of the disabled legs were not updated after being disabled (i.e., after 15 s). They remained the last values before being disabled.

Figure S6 Adaptation of the sensory feedback connection of each limb (α_*i*_) of the six-legged robot during 30 s of simulation, where two limbs were disabled after 15 s. A video showing an example of self-organized locomotion of the six-legged robot can be viewed at: www.manoonpong.com/Frontiers2020/6legsDamage.mp4. Note that the sensory feedback connections of the disabled legs were not updated after being disabled (i.e., after 15 s). They remained the last values before being disabled.

Figure S7 Adaptation of the sensory feedback connection of each limb (α_*i*_) of the eight-legged robot during 30 s of simulation, where three limbs were disabled after 15 s. A video showing an example of self-organized locomotion of the eight-legged robot can be viewed at: www.manoonpong.com/Frontiers2020/8legsDamage.mp4. Note that the sensory feedback connections of the disabled legs were not updated after being disabled (i.e., after 15 s). They remained the last values before being disabled.

Figure S8 Adaptation of the sensory feedback connection of each limb (α_*i*_) of the 20-legged robot during 30 s of the simulation, where six limbs were disabled after 15 s. A video showing an example of self-organized locomotion of the 20-legged robot can be viewed at: www.manoonpong.com/Frontiers2020/20legsDamage.mp4. Note that the sensory feedback connections of the disabled legs were not updated after being disabled (i.e., after 15 s). They remained the last values before being disabled.

Figure S9 A comparison of gait formation between adaptive and fixed sensory feedback strength in the six-legged robot. **(A,B)** Using adaptive sensory feedback strength proposed in this study. **(C,D)** Using fixed sensory feedback strength. The value of the fixed sensory feedback strength was set to 0.02 in all limbs, which is the average value to which the adaptive sensory feedback strength converges under the intact condition. The red lines in **(B,D)** indicate the periods that the gaits were formed. The gaits were formed within around 5 s for the adaptive sensory feedback strength **(B)** and around 7 s for the fixed sensory feedback strength **(D)**. Green areas represent stance phases, while white areas correspond to swing phases. After amputation occurs, the state of the disabled limbs is represented in gray. It can be seen that the robot with the adaptive sensory feedback strength could adapt its remaining legs to stay in the stance phase longer for stable locomotion compared to the one with the fixed sensory feedback strength.

Video S1 The video shows an example of self-organized locomotion of the four-legged robot.

Video S2 The video shows an example of self-organized locomotion of the six-legged robot.

Video S3 The video shows an example of self-organized locomotion of the eight-legged robot.

Video S4 The video shows an example of self-organized locomotion of the 20-legged robot.

Video S5 The video shows an example of adaptation to leg damage of the four-legged robot.

Video S6 The video shows an example of adaptation to leg damage of the six-legged robot.

Video S7 The video shows an example of adaptation to leg damage of the eight-legged robot.

Video S8 The video shows an example of adaptation to leg damage of the 20-legged robot.

Video S9 The video shows robot locomotion with different learners.
