# Supplementary figures and images for: General Distributed Neural Control and Sensory Adaptation for Self-Organized Locomotion and Fast Adaptation to Damage of Walking Robots
Source: Front Neural Circuits. 2020 Aug 17;14:46. doi: 10.3389/fncir.2020.00046 (PMC7461994; doi:10.3389/fncir.2020.00046)

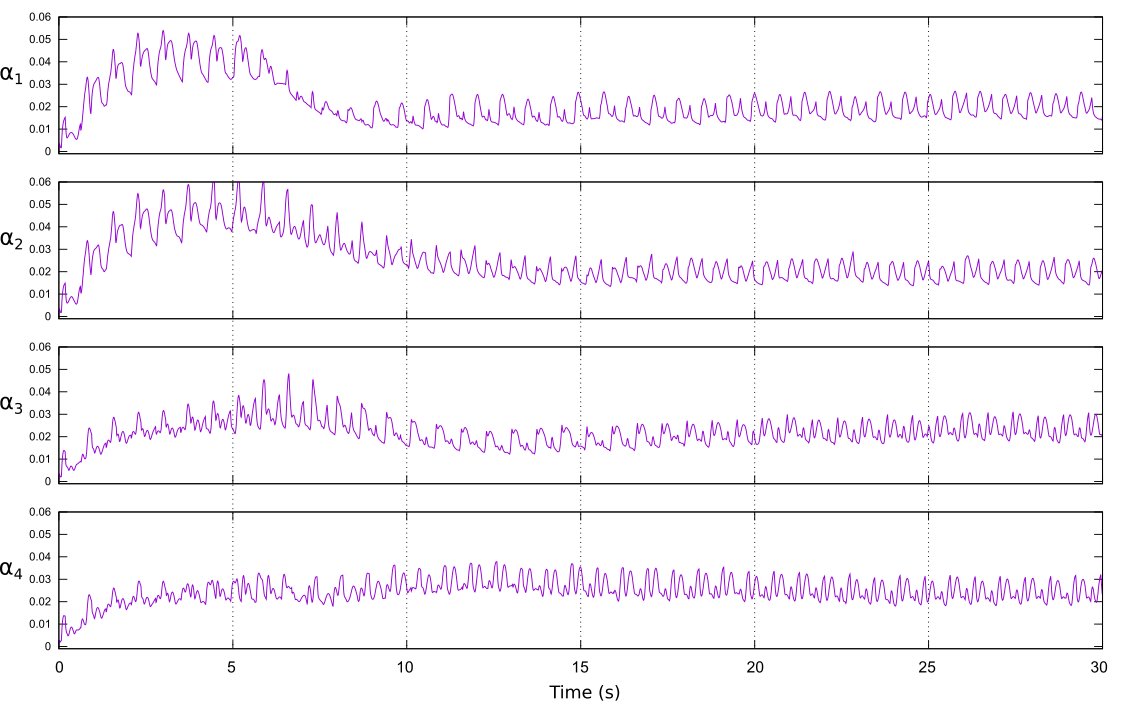

Supplement: Figure S1 — Adaptation of the sensory feedback connection of each limb (αi) of the four-legged robot during 30 s of simulation. A video showing an example of self-organized locomotion of the four-legged robot can be viewed at: www.manoonpong.com/Frontiers2020/4legs.mp4. [file Image_1.TIFF]

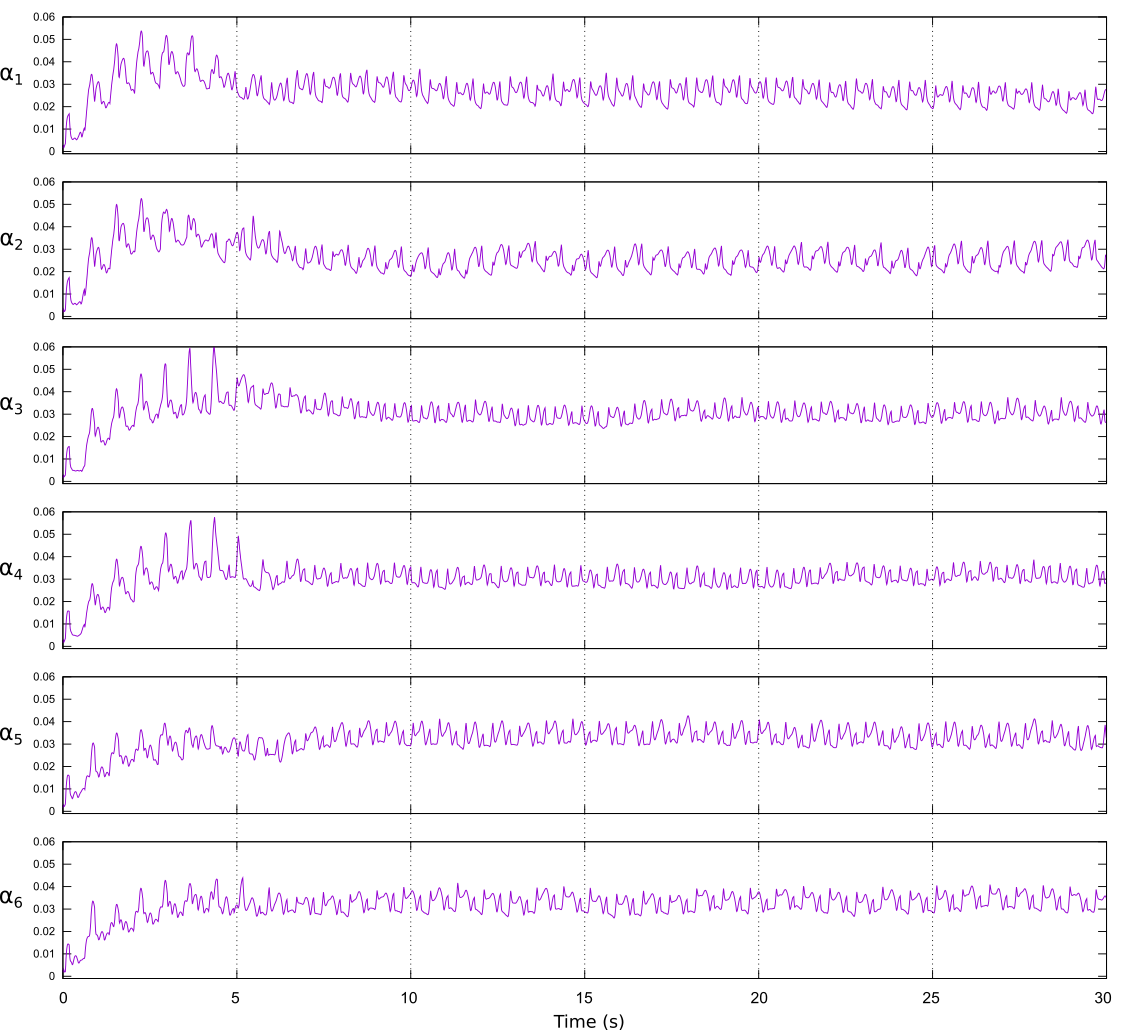

Supplement: Figure S2 — Adaptation of the sensory feedback connection of each limb (αi) of the six-legged robot during 30 s of simulation. A video showing an example of self-organized locomotion of the six-legged robot can be viewed at: www.manoonpong.com/Frontiers2020/6legs.mp4. [file Image_2.TIFF]

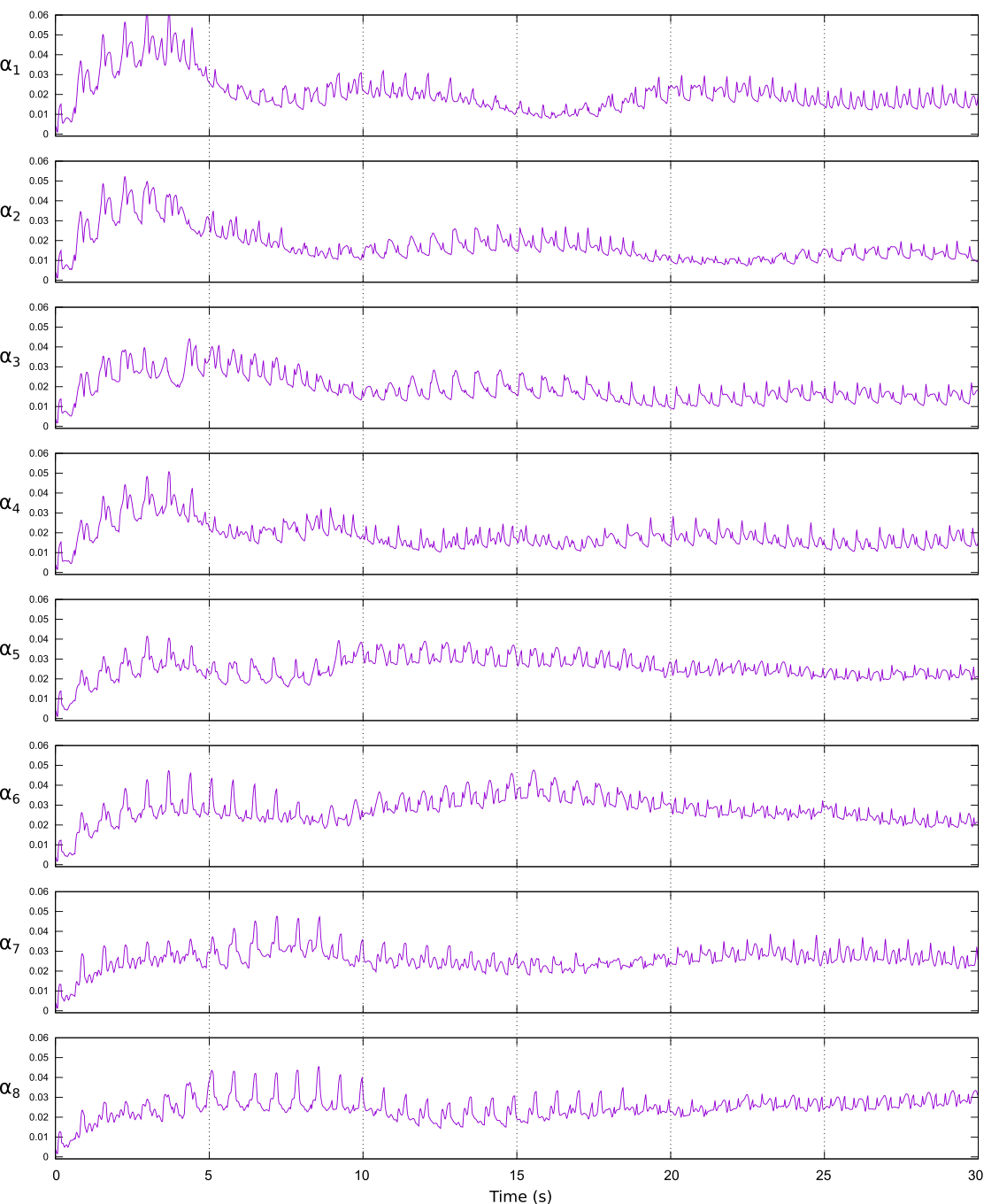

Supplement: Figure S3 — Adaptation of the sensory feedback connection of each limb (αi) of the eight-legged robot during 30 s of the simulation. A video showing an example of self-organized locomotion of the eight-legged robot can be viewed at: www.manoonpong.com/Frontiers2020/8legs.mp4. [file Image_3.TIFF]

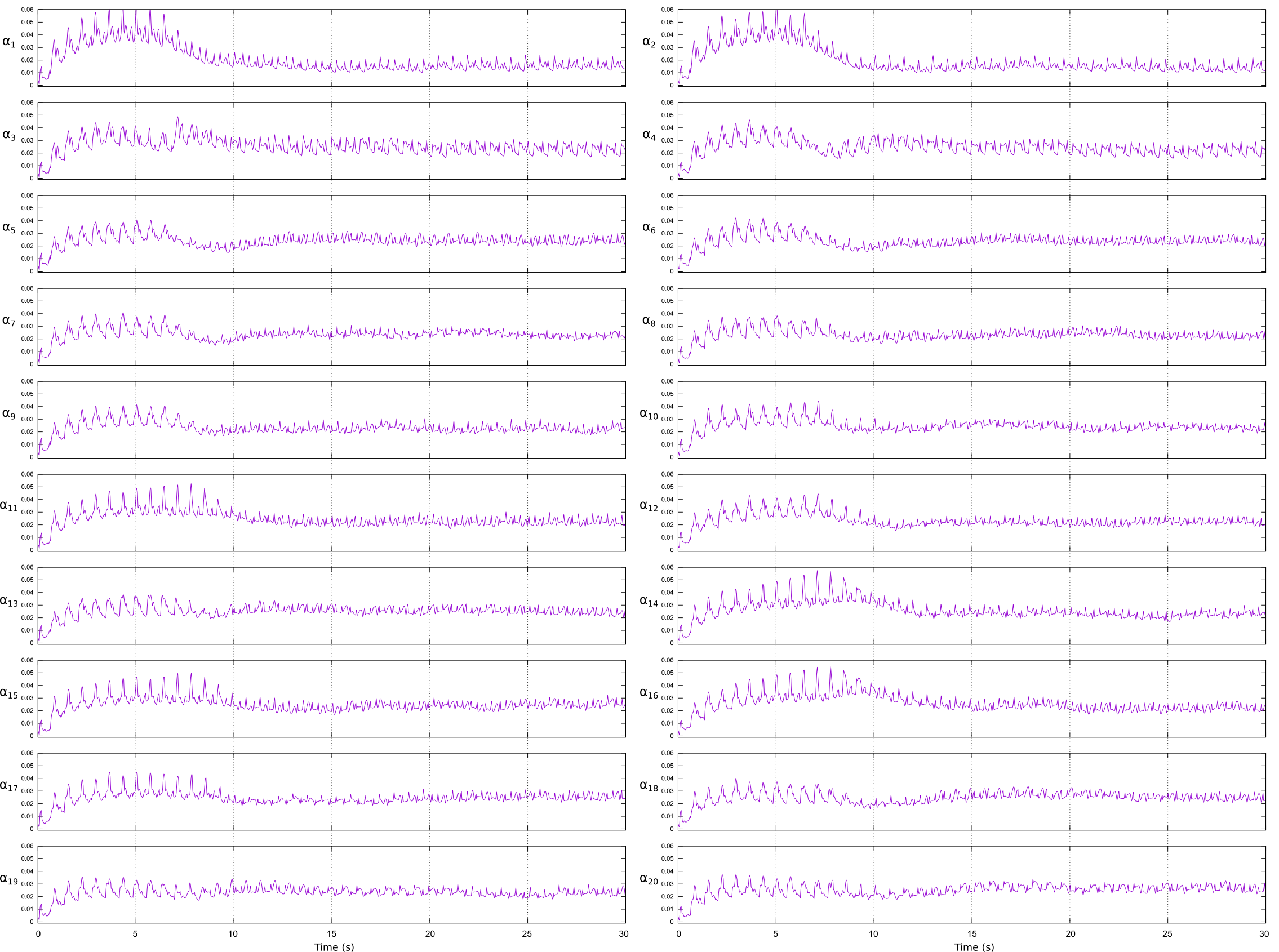

Supplement: Figure S4 — Adaptation of the sensory feedback connection of each limb (αi) of the twenty-legged robot during 30 s of simulation. A video showing an example of self-organized locomotion of the twenty-legged robot can be viewed at: www.manoonpong.com/Frontiers2020/20legs.mp4. [file Image_4.TIFF]

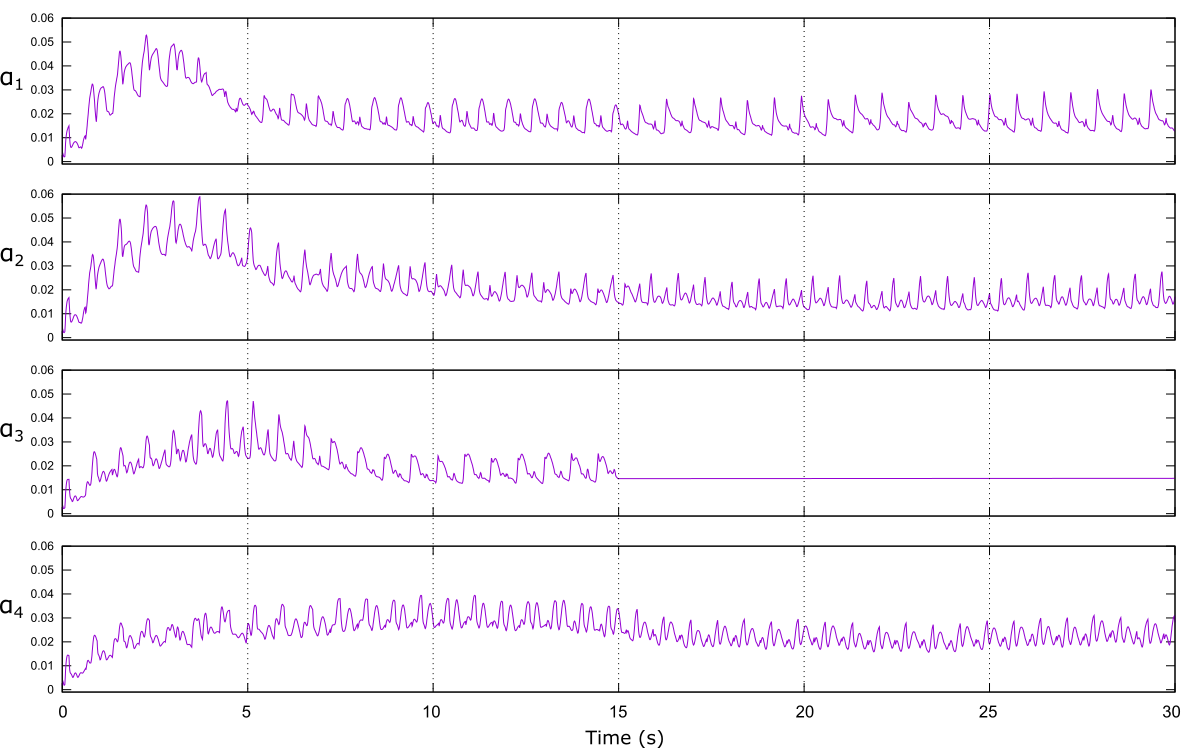

Supplement: Figure S5 — Adaptation of the sensory feedback connection of each limb (αi) of the four-legged robot during 30 s of simulation, where one limb was disabled after 15 s. A video showing an example of self-organized locomotion of the four-legged robot can be viewed at: www.manoonpong.com/Frontiers2020/4legsDamage.mp4. Note that the sensory feedback connections of the disabled legs were not updated after being disabled (i.e., after 15 s). They remained the last values before being disabled. [file Image_5.TIFF]

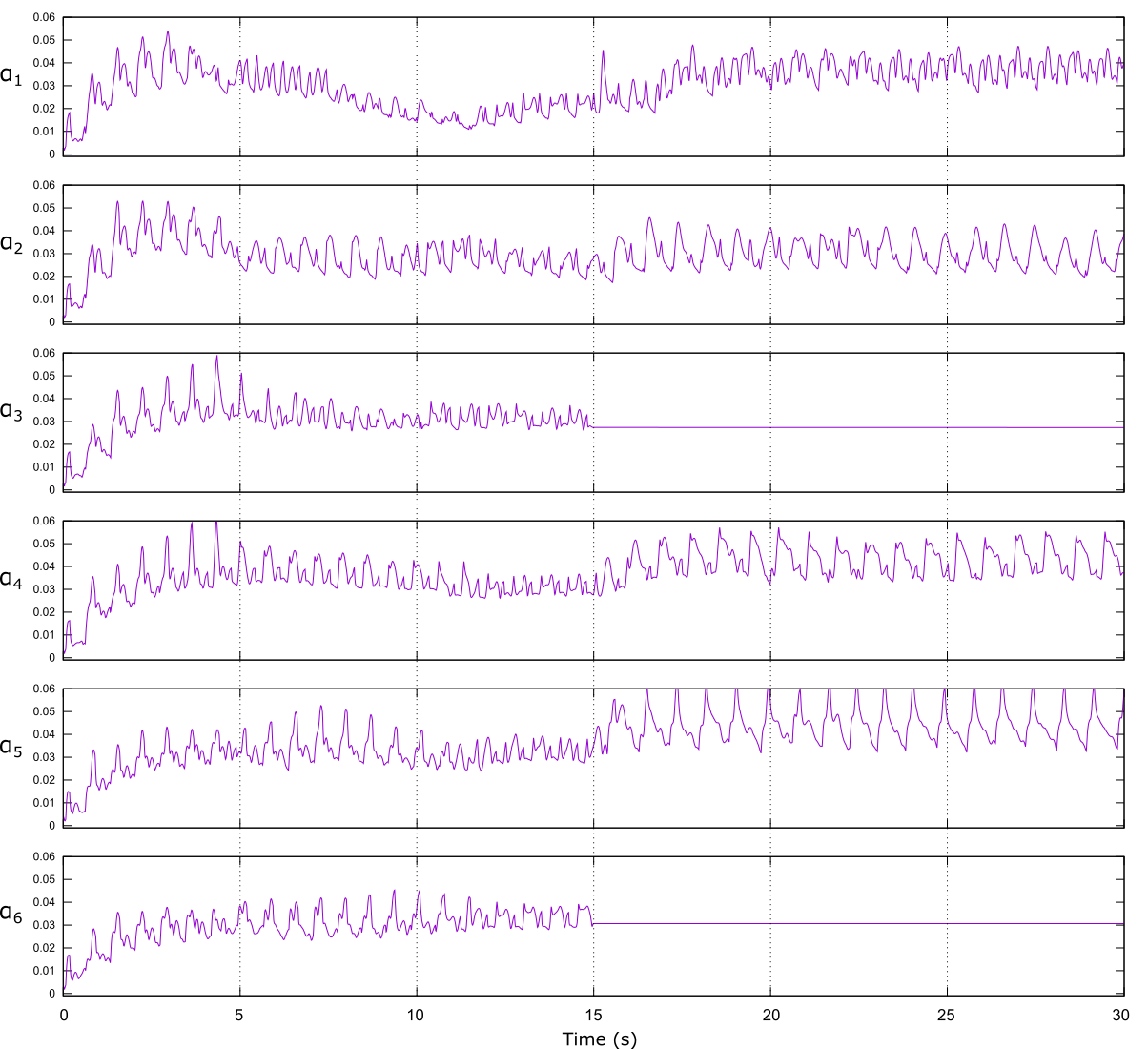

Supplement: Figure S6 — Adaptation of the sensory feedback connection of each limb (αi) of the six-legged robot during 30 s of simulation, where two limbs were disabled after 15 s. A video showing an example of self-organized locomotion of the six-legged robot can be viewed at: www.manoonpong.com/Frontiers2020/6legsDamage.mp4. Note that the sensory feedback connections of the disabled legs were not updated after being disabled (i.e., after 15 s). They remained the last values before being disabled. [file Image_6.TIFF]

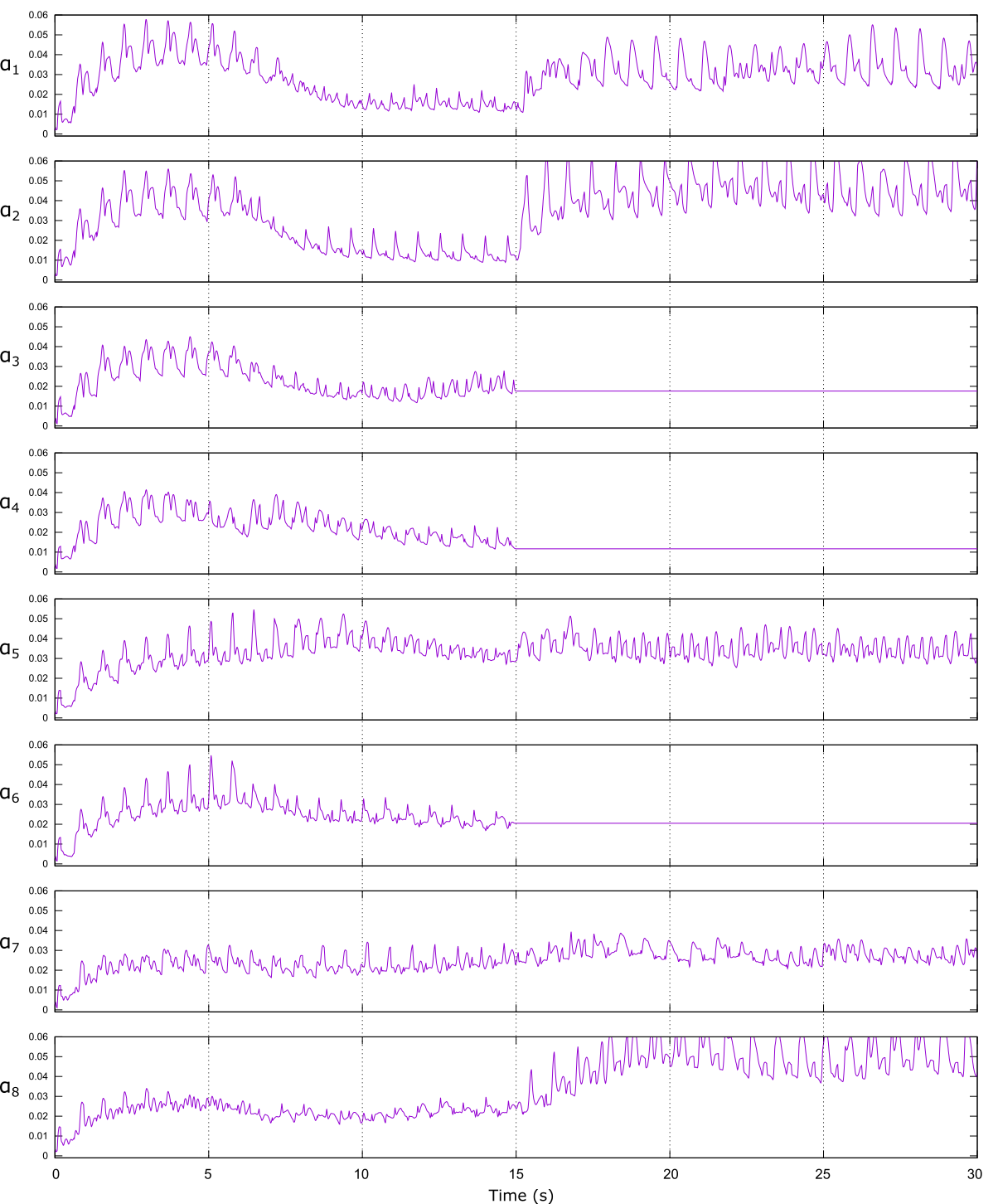

Supplement: Figure S7 — Adaptation of the sensory feedback connection of each limb (αi) of the eight-legged robot during 30 s of simulation, where three limbs were disabled after 15 s. A video showing an example of self-organized locomotion of the eight-legged robot can be viewed at: www.manoonpong.com/Frontiers2020/8legsDamage.mp4. Note that the sensory feedback connections of the disabled legs were not updated after being disabled (i.e., after 15 s). They remained the last values before being disabled. [file Image_7.TIFF]

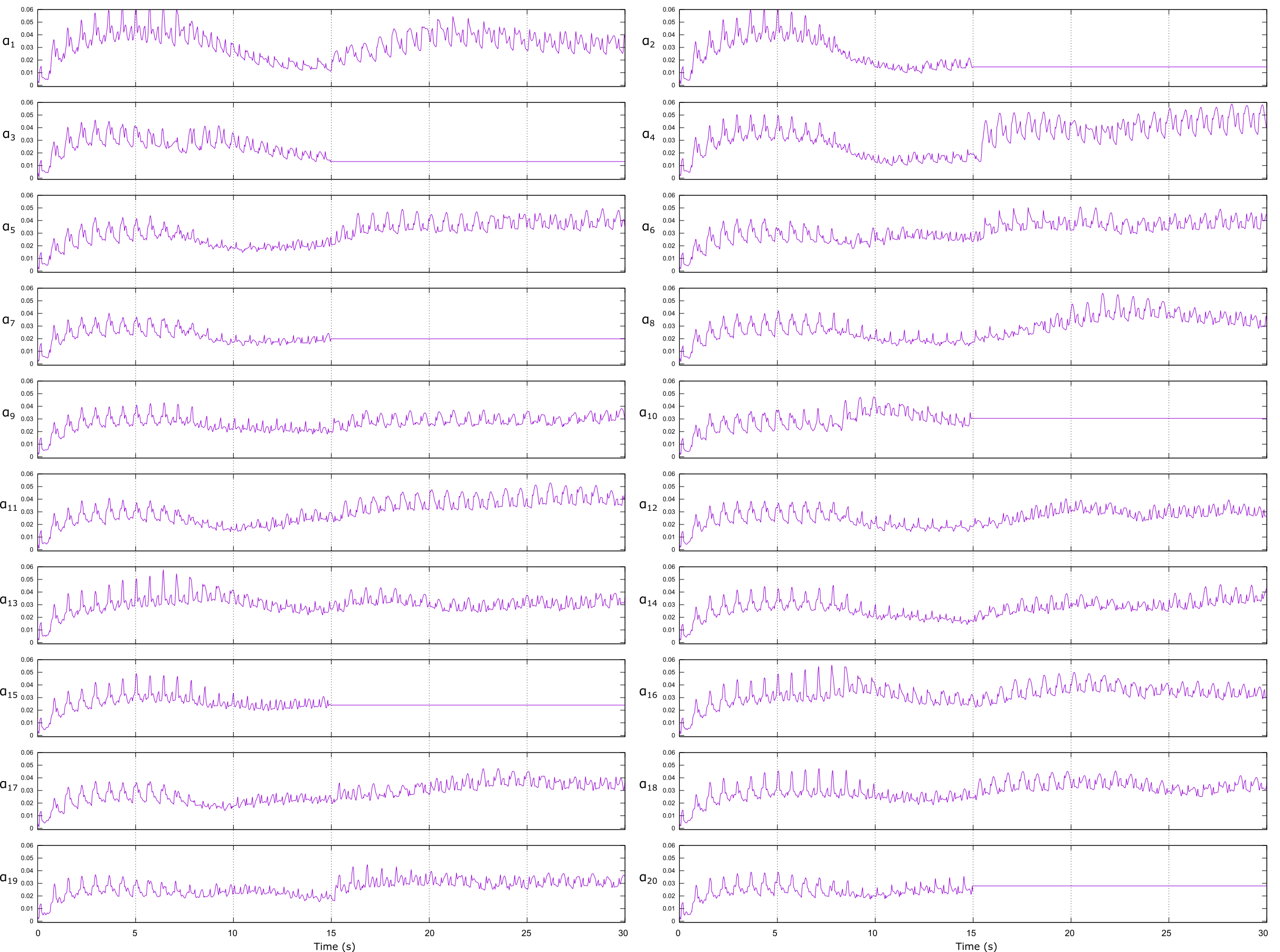

Supplement: Figure S8 — Adaptation of the sensory feedback connection of each limb (αi) of the 20-legged robot during 30 s of the simulation, where six limbs were disabled after 15 s. A video showing an example of self-organized locomotion of the 20-legged robot can be viewed at: www.manoonpong.com/Frontiers2020/20legsDamage.mp4. Note that the sensory feedback connections of the disabled legs were not updated after being disabled (i.e., after 15 s). They remained the last values before being disabled. [file Image_8.TIFF]

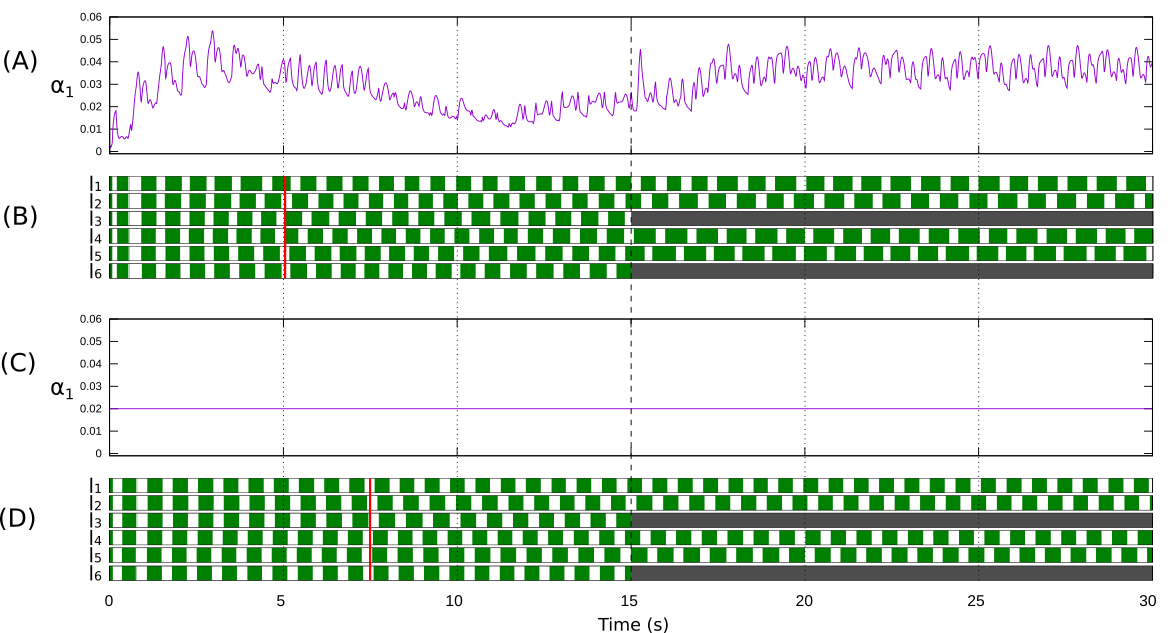

Supplement: Figure S9 — A comparison of gait formation between adaptive and fixed sensory feedback strength in the six-legged robot. (A,B) Using adaptive sensory feedback strength proposed in this study. (C,D) Using fixed sensory feedback strength. The value of the fixed sensory feedback strength was set to 0.02 in all limbs, which is the average value to which the adaptive sensory feedback strength converges under the intact condition. The red lines in (B,D) indicate the periods that the gaits were formed. The gaits were formed within around 5 s for the adaptive sensory feedback strength (B) and around 7 s for the fixed sensory feedback strength (D). Green areas represent stance phases, while white areas correspond to swing phases. After amputation occurs, the state of the disabled limbs is represented in gray. It can be seen that the robot with the adaptive sensory feedback strength could adapt its remaining legs to stay in the stance phase longer for stable locomotion compared to the one with the fixed sensory feedback strength. [file Image_9.TIFF]
